# The colonial response to the development of disease in Ghana and Côte d’Ivoire (ca. 1900-1955): A comparative analysis of British and French colonial health policies

**DOI:** 10.1371/journal.pone.0329713

**Published:** 2025-08-14

**Authors:** Arlinde C. E. Vrooman

**Affiliations:** Tilburg School of Economics and Management, Tilburg University, Tilburg, the Netherlands; University of South Florida, UNITED STATES OF AMERICA

## Abstract

Using a newly constructed dataset of official morbidity figures based on colonial medical reports, this article studies the British and French colonial response to the development of fourteen selected diseases in colonial health care facilities in Ghana and Côte d’Ivoire from circa 1900–1955. Yaws and malaria are shown to have received colonial attention due to their relatively high incidence in the facilities, while other diseases were deemed important for reasons other than the number of cases treated (sleeping sickness, yellow fever, smallpox). Despite similar forces surrounding colonial decision-making (such as the expansion of the colonial health care networks, population growth and the development of Western medicine), the British and French colonial response developed differently for part of the selected diseases (including sleeping sickness, measles and dysentery). For five commonly prevalent diseases (leprosy, dysentery, measles, gonorrhoea and syphilis) in Ghana and Côte d’Ivoire, the results in this article suggest that as of the 1930s, French colonial policymakers recognised their threat, while the British failed to do so sufficiently. A second new dataset of colonial vaccination campaigns (for ca. 1900–1955) formed the basis of an analysis of this aspect of the colonial response outside health care facilities. It finds that several diseases (including yellow fever) were addressed – more so in Côte d’Ivoire than in Ghana – but that smallpox vaccination campaigns trumped all others. The findings of this analysis indicate that extensive smallpox campaigns occurred earlier in both countries than previously suggested by the literature, and that the French colonial administration imposed a more elaborate programme.

## 1. Introduction

West Africa became known as the ‘white man’s grave’ in the 17^th^ and 18^th^ centuries [1]. Its climate was considered inhospitable to Europeans, and high mortality rates among settlers were common. Malaria and yellow fever were the principal causes of death for settlers in West Africa. The continent itself has been afflicted by disease for centuries – often worsened by its tropical climate [2]. This disease environment continues to pose a large burden on sub-Saharan African (hereafter: African) societies to this day [3]. However, until the 19th century, the local disease environment was relatively stable, because of a relatively low population growth, low contact among population groups and little technological progress in transportation [2].

This situation changed greatly when European countries started to colonize the continent during the second half of the 19^th^ century. On the one hand, some of the practices and policies of the colonial powers facilitated the spread of diseases by increasing the movement of persons across the continent: the introduction of new modes of transport, the intensification of trade among regions, and the imposition of forced labour and forced migration [2,4,5]. On the other hand, colonial rule also changed the medical environment in Africa. The rinderpest epidemic of the late 19^th^ century, sleeping sickness epidemics, and outbreaks of other diseases (including river blindness) during the early period of colonial rule formed significant threats to health in the newly established colonies. In response to the disease environment, missionaries and doctors introduced Western medical knowledge and technology, and colonial administrators invested in health care by setting up urban hospitals, although these were at first mainly aimed at serving Europeans [2,4,5]. In an attempt to manage the local disease burden, colonial authorities also launched medical interventions. They set up tropical medical schools in Europe, where Africa’s major infectious diseases and their potential treatments were studied [4,6]. The results of these studies were translated into medical interventions in African societies during the first half of the 20^th^ century, often in the form of colonial medical campaigns that treated and vaccinated the population against certain infectious diseases (such as sleeping sickness, smallpox, river blindness, tuberculosis).

A broad literature has studied different aspects of the relationship between disease, health care and colonial rule in Africa. For example, Hartwig and Patterson, Kjekshus, and more recently Alsan, and Depetris-Chauvin and Weil investigate the disease environment in Africa and changes posed by colonial rule [2,7–9]. In addition, Patterson, Headrick, and Lowes and Montero, amongst others, analyse colonial health care in several specific African countries [5,10,11]. Other influential works include Beck, Vaughan and Schneider (see Section 3 for more) [12–14]. The aim of this article is to examine the relationship between the development of disease and colonial rule in a specific context, by answering the following research question: to what extent and how did British and French colonial administrations in Ghana and Côte d’Ivoire respond to developments in official morbidity figures? To answer this question, this article pairs newly constructed quantitative datasets on official morbidity and vaccination figures during the first half of the 20^th^ century with a historical analysis of colonial medical reports. This analytical frame is grounded in a detailed reading of archival records, supplemented with discussion of secondary sources whenever relevant. In doing so, the article draws on the strengths of comparative historical analysis to identify to what extent shifts in official morbidity figures aligned, or did not align, with colonial health policy or interventions. It is also grounded in the idea that colonial responses were shaped not only by official medical data, but also by institutional considerations and colonial ideologies.

The choice of countries is motivated by two reasons. Firstly, Ghana and Côte d’Ivoire are neighbouring West African countries with a different colonial history. While Ghana was colonized by Great Britain until 1957, Côte d’Ivoire belonged to the colony of French West Africa (*Afrique Occidentale Française*, AOF; which also included present-day Benin, Burkina Faso, Guinea, Mauritania, Mali, Niger and Senegal) until gaining independence in 1960. In terms of conditions such as geography and climate, the countries were otherwise relatively alike [15]. These factors make for an especially interesting setting to comparatively analyse British and French colonial responses to the development of diseases. Secondly, the colonial records on both Ghana and Côte d’Ivoire are quite extensive.

Apart from covering the majority of the colonial period, the selected era forms an interesting setting for this study due to three topics discussed in previous literature. These topics become relevant during the first half of the 20^th^ century, overlapping with this article’s period of study. Firstly, the germ theory of disease became widely accepted by the end of the 19^th^ century [16]. It replaced previous perceptions on the cause of disease, such as ‘bad air’, with the notion that germs could lead to diseases [17]. A period of rapid discoveries of transmission mechanisms of disease ensued soon after. Secondly, modern medicine had developed in Europe during the 19^th^ century, due to technological advancements such as anaesthetics, antiseptics and the stethoscope [18]. These advances provided the necessary building blocks for further advances in the 20^th^ century, such as synthetic drugs and chemotherapy. Colonial rule also instilled a new interest in medical research that studied issues in tropical medicine. Lastly, the international epidemiological transition started during the final decades of the period under consideration. Beginning in the 1940s, poorer countries experienced quick increases in life expectancy as a result of rapid global spread of new technologies and practices [19].

This article’s findings suggest that the responses of British and French colonial administrators were motivated by various reasons, depending on the type of disease. Part of the selected diseases (yaws and malaria) received colonial attention because of their relatively high incidence. Other diseases (yellow fever, sleeping sickness and smallpox) took the forefront in colonial policy for reasons unrelated to the number of cases treated. The British and French colonial response to yellow fever and sleeping sickness were the consequence of the diseases’ effects on Europeans in Ghana and Côte d’Ivoire, while the availability of smallpox vaccinations spurred on its importance in both administrations’ health policy in the two countries. The remainder of the selected diseases received less priority from British and French colonial policymakers. The analyses of leprosy, dysentery, measles, gonorrhoea and syphilis indicate that the French colonial administration in Côte d’Ivoire was comparatively faster in translating colonial policy shifts towards African development during the 1920s and 1930s into an increased number of patients treated in colonial health care facilities. The development of the number of cases treated (per capita) for these diseases in Ghana deviates, which is suggested to (partly) be the result of a sluggish response by the British colonial administration. Finally, an analysis of a new dataset on colonial vaccination campaigns established that several diseases were addressed through vaccination policies in both colonies. A wider range of diseases was included in these campaigns in Côte d’Ivoire compared to Ghana, but smallpox campaigns were found to be the largest in both countries. By quantifying the extent of these campaigns during the early 20^th^ century, I also show that smallpox vaccination campaigns started earlier in Ghana and Côte d’Ivoire than found in previous literature, and that French campaigns were more sizeable than British – even when accounting for differences in population sizes. In sum, this article shows that ‘the’ British and French colonial response to the development of official morbidity figures was in fact multidimensional and distinctive for the selected diseases, following general developments in policy and medical knowledge, and differences in colonial health policy. Its findings extend the literature by considering a larger scope of diseases, taking a comparative perspective, and considering the relationship between the development of morbidity within colonial health care facilities and colonial health care policy.

The remainder of this article is structured as follows. Section 2 presents the methodology and data used in the analyses. Section 3 reviews literature relevant to this article and elaborates on its contributions. Section 4 analyses and discusses patterns and trends in a selection of diseases that were treated in colonial health care facilities in Ghana and Côte d’Ivoire, and the response by the French and British colonial administrations. Moreover, Section 4 studies colonial vaccination campaigns in both countries, and focuses in particular on smallpox vaccination programmes. Section 5 summarizes the outcomes and discusses the main conclusions.

## 2. Methods and materials

The methodology of this research article consists of several elements. As is common in papers in economic history, I first review relevant strands of literature on their main findings and arguments related to this article to identify gaps in the existing literature (Section 3). In doing so, this literature review also provides necessary historical context based on the existing literature that serves as a theoretical frame and background for the article, and highlights its contribution made to the existing literature. For this purpose, I consider literature on the effect of European involvement in Africa’s disease environment, literature detailing the development and/or colonial policies for specific diseases, and literature on colonial health care provision.

Subsequently, I present and discuss the findings of my analyses, in which I combine quantitative analyses based on new data with qualitative analyses (Section 4). This article pushes the boundaries of the current literature on disease and health care under colonial rule, through an analysis of newly constructed datasets on morbidity and vaccinations. The colonial response to disease can be considered from different angles: from a broad perspective focusing on (general) policy responses to official morbidity figures, and from a more narrow view by considering formal colonial (vaccination) campaigns against particular diseases. Therefore, in this article, I first consider primary and secondary literature on colonial health policy when analysing the wider colonial response to disease patterns and trends discussed in Section 4. Next, I take a more narrow view on the colonial response to disease by analysing colonial vaccination programmes in the two countries, with a particular focus on the smallpox vaccination campaigns during this period (Subsection 4.5). For these purposes, two new datasets were compiled based on colonial medical reports. These unique datasets provide annual information on morbidity in colonial health care facilities for selected conditions, and on annual figures on the number of vaccinations, during the first half of the 20^th^ century. In the remainder of this article, colonial health care facilities are considered to be health care facilities that were administered by colonial rulers.

The Gold Coast Medical reports were used as a source to gather information on morbidity figures in Ghana [20–25]. For Côte d’Ivoire, similar information was found in colonial medical reports whenever available, and supplemented with information from annual reports on the *Service Sanitaire* and the *Rapport Annuel d’Ensemble* when necessary [26–29]. The same sources form the basis of the dataset on vaccination programmes for Ghana. For Côte d’Ivoire, the timeseries on smallpox vaccination programmes were supplemented with Schneider’s data for several years that did not report information (1942, 1945 and 1955–1957) [14]. Most of the relevant information that was available concerned country-level information, without being able to identify individual patients (neither during nor after data collection). This limitation in the level of reporting implies that it is impossible to differentiate at a finer grained level (geographic origin of patients, among regions, among different types of patients, etc.). Sources for Ghana were accessed on March 30, 2020, and for Côte d’Ivoire on October 1, 2020. As the data is taken from historical records dating back to ca. 1900–1955, that were anonymized and included only overall aggregated figures (no individual data), no ethics committee approval was needed. An overview of the sources used is available in the Data Appendix (S24 Text).

In general, disease tables for Ghana were reported consistently throughout the colonial medical reports. Figures on mortality per disease were also registered. Few changes in style and level of reporting occurred, as a standardized format was used. Most notable is a change in separating outpatients from inpatients for 1911–1915 and 1928–1955. Information on disease cases for Côte d’Ivoire was less consistent. Issues concern changes in level of reporting, as well as variation in the level of health care facilities included, in addition to missing information within reports due to omission of specific disease accounts in some years. Additionally, only a few years included data on mortality figures. As a result, a comparative analysis of official mortality could not be fully incorporated in this article. The Data Appendix (S24 Text) elaborates on the reasons for discarding mortality figures from the analysis and provides a more detailed discussion of the data that is available. Moreover, annual reports on Côte d’Ivoire were not always available or complete, leading to no information for several years (1908, 1910–1912, 1915–1923, 1926–1927, 1934).

A selection of the reported diseases was necessary to compare patterns and trends between Ghana and Côte d’Ivoire. S1 Fig on jaundice highlights some of the issues in analysing official data and illustrates why a selection was needed. In Ghana, jaundice data is incomplete (especially after 1938), while no cases are recorded for Côte d’Ivoire. The reasons for missing data, be it an absence of cases, a reporting error, or something else, are unclear. Similar gaps exist for other diseases, which were therefore excluded from the selection. Data availability formed the primary selection criterion, but comparability between the two countries also played a role. This selection criterion led to the exclusion of skin diseases, as format differences in the reports prevented the construction of a comparable variable. Moreover, importance formed a selection criterion; both in terms of importance attributed to the disease during colonial rule as illustrated by policy debates and/or measures, and ‘absolute’ importance as signified by substantial patient figures reported in the colonial documents. Lastly, diseases that form part of existing debates in the current (secondary) literature (e.g., sleeping sickness) were also included in the dataset. in the final dataset includes the number of cases treated within colonial health care facilities for beriberi, chickenpox, dysentery, gonorrhoea, influenza, leprosy, malaria, measles, sleeping sickness, smallpox, syphilis, tuberculosis, yaws, and yellow fever.

Concerning vaccination programmes, all available information on the number of vaccines administered by colonial health care was transcribed from the medical reports. This included information drawn from overview tables, but also in-text mentions of numbers of vaccines provided for specific diseases. The number of diseases that was reported varied between the two colonies, but the medical reports included information on vaccines against multiple diseases, such as smallpox, yellow fever and plague (S19 Table). Some of the vaccines were provided as part of larger vaccination campaigns, for instance against smallpox. The findings (Section 4.5) contribute to the existing literature (Section 3) by taking a comparative frame that allows me to review the similarities and differences in British and French colonial vaccination policy. Schneider’s study of smallpox vaccinations across Africa is pioneering, but lacks systematic data before the 1920s [14]. This article’s new data pushes the analysis back to the early 20^th^ century (1902–1957) for Ghana and Côte d’Ivoire. It also uses a different methodological approach, transforming the timeseries into per capita figures instead of absolute numbers using the African population estimates by Frankema and Jerven to be able to compare between the two countries [30]. Data for alternative denominators such as the number of patients was not available, hence the choice to include population figures. This transformation allows for a clearer comparison between the two countries, revealing differences in both the intensity and timing of smallpox vaccination campaigns in Section 4.5.

Scholars have mentioned various issues concerning data in reports from the colonial period. For census and parish registers, Walters discusses that this type of data suffers from potential underestimation, censoring and selection effects [31]. On colonial finances, Westland demonstrates that British colonial price data in the Blue Books is of fluctuating accuracy [32]. Research by e.g. Frankema and Gardner has established data on colonial expenditures as generally accepted, in part due to a lack of alternative data [33,34].

Data on patients, as used in this article, suffers from issues similar to those encountered in population research. Specifically, selection effects occur. The colonial medical reports only record diseases on patients who were treated within colonial health care facilities. Persons who lacked access to colonial health care facilities, or sought treatment outside of these facilities, will not show up in the disease tables mentioned in the colonial medical reports. Since no other sources are available to fully assess the groups of patients outside colonial health care facilities, the analyses and findings in this article can only quantitatively assess the colonial response to patients treated by colonial health care facilities. Particular care is taken in emphasizing this caveat when interpreting results. Moreover, cases and mortality figures may be linked to the geographic reach of the colonial health care system, its ability to treat specific diseases, and its reputation. Those who were too ill to travel, or knew, or believed that the colonial health care facilities would not provide them with a (sufficient) cure, would not have arrived in colonial health care facilities and therefore do not show up in the records used in this article. Based on the colonial reports, it remains unclear how large this group of potential patients would have been, which makes it difficult to assess the level of underestimation as a result of their absence in the figures.

**Fig 1:**
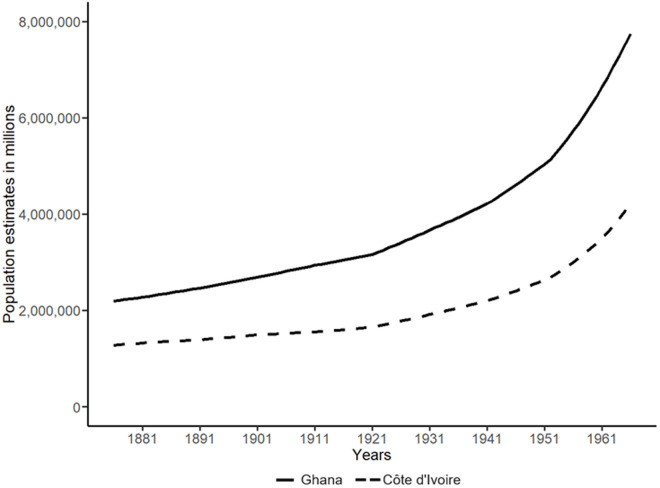
Development of population estimates in Ghana and Côte d’Ivoire, 1875-1965. Data source: [30].

Another issue to keep in mind is that population increased considerably in Ghana and to a lesser extent in Côte d’Ivoire during colonial rule, as shown in Fig 1. Population growth itself is a contributing factor to the spread of disease, especially if growth occurs in and/or leads to densely populated regions, by increasing contact between individuals and thereby facilitating easier transmission of diseases. However, and perhaps more importantly, by considering per capita cases for comparability between the countries, changes in the development of disease cases (per capita) over time may be (partly) driven by changes in population figures. Ideally, one would solve this issue by selecting a better denominator, for instance the number of patients that were treated within colonial health care facilities. Unfortunately, patient data on Côte d’Ivoire in particular is reported sparsely in the colonial medical reports, and does not allow for the construction of a timeseries on the development of the total number of patients in colonial health care facilities over time [35]. The bias stemming from the use of per capita figures would be mitigated if the assumption that increases in population lead to a similar growth in the number of cases treated holds. This would require a) a link between population growth and an additional uptake of colonial health care and b) an adaptive colonial health care system that could accommodate for increasing patient figures. To some extent, evidence in the literature suggests that the expansion of the colonial health care systems could be demand-driven (see, e.g., Lasker or Rouanet), especially in the later decades when colonial health policy widened its focus beyond European care [36,37]. However, we also know that in general, the colonial health care systems were underfunded and could not meet the demands of the population [10,36]. Hence, whether this assumption holds is up for debate.

There are various reasons why observations of the number of cases for specific diseases may rise (see also S32 Text). Firstly, the development of Western medicine and medical knowledge can lead to increases in the number of observations for a specific disease, as the application of these changes in the medical environment caused more patients to arrive in colonial health facilities, or patients were specifically sought after to implement a particular treatment or preventive method. Although this effect is not mitigated, this article is aware of these changes, and includes them in the interpretation of the development of diseases whenever possible. Observations also rise when additional health care facilities are opened, because more patients could be treated. The number of colonial health care facilities increased considerably as the colonial health care systems in Ghana and Côte d’Ivoire developed [35]. Consequently, one should be careful when interpreting changes in the number of cases. Lastly, colonial recordkeeping could have improved over time. As discussed by Rouanet, increases in the number of cases for a disease do not automatically signal an increase in the overall prevalence of the disease, but could also be the result of improvements in the documentation of the disease [37]. The relative consistency of the format in which diseases were reported, especially for Ghana, suggests that this potential issue could be limited in this article’s cases.

Finally, the dataset on vaccination programmes suffers from underestimation. The 1903 Gold Coast Medical already recognized this issue, and ascribes it to persons failing to report for observation after starting the vaccination programme [21]. This attrition bias likely also holds for the vaccination data on Côte d’Ivoire. Selection problems also occur, but on a different scale than for morbidity figures. The provision of vaccinations was not limited to colonial health care facilities, but also consisted of campaigns that visited various locations throughout the colonies over time. As a result, treatment was available to those who were close to these locations, rather than to those who had access to and/or could travel to colonial health care facilities. The bias derived from the use of per capita figures and alternative factors contributing to changing trends also apply here.

## 3. Literature review

Before the onset of colonial rule, a wide variety of diseases afflicted African societies, and especially West Africa’s disease environment was notorious in the 17^th^ and 18^th^ century for its adverse effects on settlers [1]. Part of the literature on the relationship between disease in Africa and the effect of European involvement starts in this pre-colonial period. Although it is not the focus of this article, I briefly review this strand of literature to provide some context for this period. It is important to note here that this section’s focus on literature detailing the interaction between Africa’s disease environment and European involvement should not be considered as an indication that traditional medicine was not present or should be dismissed. Herbert, for instance, shows that traditional inoculation practices against smallpox in Africa started well before colonial efforts against smallpox were launched, dating back to at least the 18th century [38]. Traditional healing practices included herbal remedies applied at home, and consultations with indigenous health care practitioners for afflictions, diseases and other events that required medical assistance [4,39]. Despite facing resistance from European colonizers during the 19^th^ and 20^th^ century, traditional medicine continued to persist in Africa under colonial rule [4,10,40].

European involvement prior to the Scramble for Africa influenced the local disease environment. Curtin, for example, links the trans-Atlantic slave trades to increased mortality figures, through their effect on changes in the disease environment of African countries [41]. The literature also shows that European medical involvement started before official colonial rule. In particular, Good discusses how Protestant and Catholic medical missionaries were the first to initiate the transfer of Western biomedicine in Africa, especially as of the second half of the 19^th^ century [42]. Webb includes a discussion of the early attempts by Europeans to influence the disease environment in African countries to shield themselves against the disease [43].

Colonial rule led to changes in the local disease environment, and introduced new methods to address the burden it posed on the population, as put forward in the case of Tanganyika by Kjekshus, and for Africa in general by Hartwig and Patterson [2,7]. The literature considers several diseases with a substantial impact on the development of health (care) during colonial rule. Part of the debate focuses on sleeping sickness epidemics and the colonial response. Initial works focused on specific colonies, for example by Domergue on Côte d’Ivoire and Lyons on Belgian Congo [6,44]. Taking a comparative perspective, Worboys considers the colonial response to early sleeping sickness epidemics in East and Central Africa (1900–1914) [45]. For the same region during the colonial period (until World War II (WWII)), Headrick finds that although many colonies faced similar epidemics, the response differed per colonizer. Generalizing, the French approach focused on diagnosing and treating sleeping sickness, while the British approach attempted to prevent the disease by vector control [46]. Similar to the French, the Belgian colonial administration focused on containing the spread of disease through measures like lockdowns and medical campaigns, while the Portuguese authorities – limited by financial constraints – only focused on treatment campaigns. Lowes and Montero empirically show that such sleeping sickness campaigns in former French colonies in Central Africa had a lasting impact on current trust in medicine: areas that were visited by colonial sleeping sickness campaigns have a lower level of trust in medicine [11]. They argue that the violent nature of the French campaigns, where persons were forced to participate (even at gun point), explains this result.

The considerable research attention paid to the relationship between colonial rule and sleeping sickness is not reciprocated for other diseases in the current debates, even though many other poignant afflictions were, and continue to be, present in African countries. Nonetheless, certain other diseases have also received some attention in the literature studying disease under colonial rule. The outbreak of rinderpest in the late 19^th^ century, which decimated the cattle herd in parts of Africa and required the response of colonizers while colonial health systems were still in their start-up phase are linked to the sleeping sickness epidemics in the early 20^th^ century through its effect on the disease environment [46]. Schneider provides an excellent overview of smallpox vaccination programmes implemented by colonial authorities in Africa [14]. He finds that the success of these campaigns varied. In some countries, colonial authorities were successful at controlling smallpox, while in other countries smallpox was only eradicated after colonial rule, once international cooperation and different systems of vaccination were employed. The literature also shows that colonial rule did not always respond to developments of disease in a timely fashion. Patterson discusses how colonial medical services failed to observe the considerable effect of river blindness (or onchocerciasis) on the population of Northern Ghana until the 1940s [47]. For the same region, Bannister argues that the British colonial administration was late to recognize the urgency of increased prevalence of both sleeping sickness and river blindness [48]. He suggests that this view changed only once economic and political developments forced British policymakers to acknowledge the threat of these diseases (in the 1930s and 1950s respectively). Moreover, Arnold describes how cholera epidemics in British India during the 19^th^ and 20^th^ century were met with a slow colonial response due to fears for political upheaval paired with financial constraints and a lack of medical knowledge [49]. This lack of response was not limited to British colonial administrations. Ngalamulume discusses the development of yellow fever in Senegal, and the slow response of the French colonial administration in the 19^th^ and early 20^th^ century [50]. Other scholars contributing to the debate on disease and colonial rule include Dawson on yaws and smallpox in colonial Kenya, Bado on leprosy, sleeping sickness and river blindness, Worboys on leprosy in British colonies, and Doyle on the pre-HIV period, to name a few [51–55]. Becker and Collignon discuss the role of 20^th^ century epidemics in West Africa on the development of colonial policy, while Dianzinga considers the same question in the context of Congo [56,57].

Apart from policies aimed at specific diseases, another part of the literature discusses how colonial administrations were concerned with general health care provision, and introduced their own health services in Africa. Their development has been discussed in detail for various regions. Patterson provides a detailed study of health and health care during colonial rule in his book on Ghana [10]. Addae also discusses colonial health care in Ghana [58]. Lasker and Domergue-Cloarec provide in-depth studies of Côte d’Ivoire, while Headrick focuses on French Equatorial Africa [5,36,59]. Studies on British East African countries are performed by Beck and Crozier, and Nkwam considers British West Africa [12,60,61]. Work by Iliffe and Greenwood considers colonial medical staff in East Africa [18,62]. Other scholars that consider colonial health care include Shapiro, Vaughan, Tilley and Rouanet [13,37,63,64]. In general, the findings of this strand of literature suggest that colonial health care provision was initially introduced to protect the health of Europeans in the colonies. After establishing a basic health care network for Europeans, and motivated by the economic exploitation of the colonies, colonial health care provision was expanded to the local population in later decades of the 20^th^ century. A body of literature on missionary health care discusses its continued importance in the available health care during colonial rule (see, e.g., Good, Vaughan, Jennings, Pringle [13,42,65,66]).

Taken together, these strands of literature inform the analytical approach adopted in this article as a theoretical frame, particularly in respect to its focus on institutional responses to disease in a comparative colonial context. However, several questions remain unanswered. The bulk of the disease literature operates in isolation, by considering one disease out of a select group of afflictions (for instance sleeping sickness). Does this focus reflect the overall importance of these diseases, or do other diseases also deserve our attention? Do the findings in the debates in the literature hold when considering a wider spectrum of diseases? Moreover, the current literature generally does not analyse the development of disease under colonial rule in a comparative context, as studies usually focus on either one country or one area colonized by the same European country. Does a difference in colonizer matter in determining the policy response to (the development of) morbidity within the colonies? A final important issue that is currently understudied in the literature on colonial health care concerns the influence of the spread of disease on the development of colonial health care policy. Scholars have paid some attention to the general role of certain large epidemics in shaping colonial health policy, including sleeping sickness, but the literature does not consider this relationship in detail. Do official morbidity figures influence the course of colonial policy, or are other factors (such as the influence of afflictions on Europeans) considered in determining health policy measures?

This article aims to form a stepping stone in studying these issues by considering the colonial response to the development of disease in a specific (comparative) context (Ghana and Côte d’Ivoire during the first half of the 20^th^ century). It pushes the boundaries of the present debates by providing new insights on the development of British and French colonial health policy in response to changes in official morbidity figures. Newly constructed datasets of unprecedented detail on morbidity figures in colonial health care facilities and colonial vaccination programmes allow me to study this relationship comprehensively and comparatively. In particular, this article contributes to the literature in six ways. Firstly, it provides a comparative perspective on the effect of colonial rule on disease and health for two Western African countries with a different colonial administration, which is generally not included in the literature on disease. Moreover, it extends the debate by considering a group of diseases that include widely studied afflictions such as sleeping sickness, but also diseases that have received less attention in the literature (e.g. measles). Thirdly, this article yields new insights in the extent and the different ways in which colonial health care policy was influenced by the development of disease, through an analysis of the British and French colonial response to official morbidity figures based on new data. Furthermore, Subsection 4.5 contributes to the literature by reviewing and comparing the entire vaccination programmes introduced by the British and French administrations based on new data gathered from colonial reports, and by extending the literature on smallpox to the early colonial period for Ghana and Côte d’Ivoire and by using a different methodology. Finally, two new datasets on morbidity and colonial vaccination programmes were constructed for the analyses in this article, that can be used in future studies on the development of disease and colonial health care in Ghana and Côte d’Ivoire, or the African region at large.

## 4. Results

In this section, I present the results from the quantitative analysis based on new official morbidity data that considers patterns in colonial health responses to fourteen selected diseases in Ghana and Côte d’Ivoire. Instead of discussing them individually, this section groups diseases thematically by how and why they received colonial attention – due to their frequency, their effect on the European population, the availability of preventive treatment – and a remaining group of the selected diseases, and finishes with an overall synthesis of the comparative analysis. The pathology of all diseases in Subsections 4.1 to 4.4 is discussed briefly in the Glossary in the Appendix (S30 Text). A detailed discussion of the patterns and trends observed in the plotted official morbidity figures (per capita) is available in the Appendix (S31 Text). S32 Text details several additional considerations and dilemma’s surrounding these analyses.

### 4.1. Most frequently treated diseases by colonial health care in Ghana and Côte d’Ivoire

#### 4.1.1. *Yaws.*

**Fig 2:**
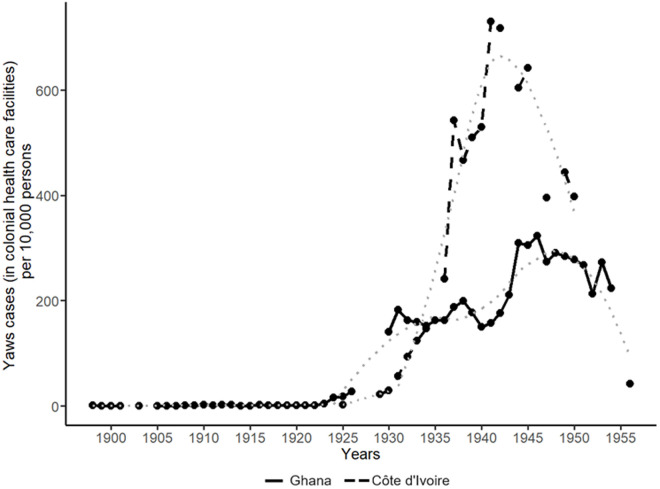
Yaws cases in colonial health care facilities per 10,000 persons, ca. 1900-1955: Ghana and Côte d’Ivoire. Note: the dotted lines represent the (curved LOESS) trends for Ghana and Côte d’Ivoire. Data source: [20–29].

By the early 20^th^ century, yaws was a common tropical disease in both Ghana and Côte d’Ivoire, affecting populations well before colonial rule [10,67–70]. Early colonial responses were hindered by a lack of effective treatments, with both traditional and Western medicines proving ineffective. In Ghana, a breakthrough came in 1920 when Dr Reindorf began using arsenical injections to treat yaws and syphilis at the venereal disease clinic in Accra, leading to a considerable and widespread uptake of treatment by the local population [10,23]. Other efforts included the introduction of sobita by Dr Hendrie in Yendi and improvements in hygiene and water provision [10,24]. Despite the positive developments in treatment availability as of the 1920s, several problems also plagued the British colonial progress against yaws in Ghana during the 1930s: high relapse rates, occasional severe side effects and a lack of medical services lagging progress in rural area. These contribute to the difference observed in Fig 2 between the two countries by the late 1930s.

Colonial health policy aimed at yaws broadened during the 1940s, translating into a rise in official yaws cases in Ghana (Fig 2). Treatment for yaws became free in 1942, resulting in an uptake of colonial health care for the disease (Fig 2) [24]. Penicillin turned out to be very effective in treating yaws (as tried in Winneba in 1944), and the drug became widely used by the colonial health services in 1953 [10,24]. A drop in patients treated in 1954 in colonial health care facilities was cautiously suggested to be linked to the recent widespread use of ‘antibiotic substances’ (Fig 2) [25]. The WHO and UNICEF’s yaws eradication program, starting in 1955, provided penicillin to Ghana, which accepted 21,000 vials for 1956 and 1957 [25]. By the 1960s, yaws had largely been eradicated in Ghana, though the disease resurfaced in the 1970s as medical priorities shifted [10,71,72].

In the 1920s, French colonial campaigns against yaws began in Côte d’Ivoire, but early efforts were hampered by the use of intravenous arsenobenzol (and its derivatives), which was difficult to administer to young children, and the limited availability of doctors who were entrusted with providing this treatment [29,35,72]. The situation improved with the introduction of Stovarsol (or acetarsol) in 1924, which could be taken orally [29]. Despite initial supply issues, the number of patients treated started to increase (Fig 2) [26].

By 1935, Stovarsol was no longer the favoured treatment in French colonial health care leading to its replacement with bismuth in 1937: after administration of Stovarsol, patients continued to suffer from yaws, some even experiencing more serious relapsing [26]. According to the French colonial medical reports, bismuth injections were accepted by the local population and showed ‘excellent results’ [26,28]. A continued effort against yaws during the 1930s and early 1940s fit within the French colonial policy shift around this period towards social development. Its necessity was further motivated by the prevalence of the disease: most of the yaws cases in French West Africa were found in Côte d’Ivoire by the early 1940s [59]. However, after WWII the capacity of the French colonial medical service declined, which is also reflected in the official morbidity figures (Fig 2). By the end of colonial rule, the WHO/UNICEF penicillin-based eradication programme led to substantial decreases in the prevalence of yaws in Côte d’Ivoire. The disease resurged in the country as of 1975, resembling Ghana’s experience [73].

#### 4.1.2. *Malaria.*

**Fig 3:**
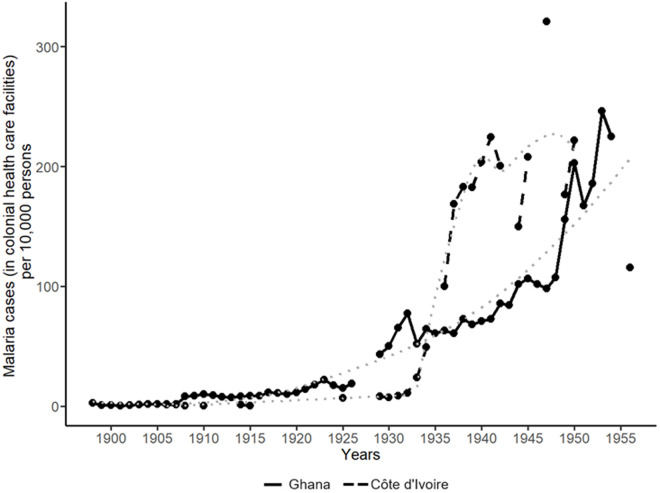
Malaria cases in colonial health care facilities per 10,000 persons, ca. 1900-1955: Ghana and Côte d’Ivoire. Note: the dotted lines represent the (curved LOESS) trends for Ghana and Côte d’Ivoire. Data source: [20–29].

Malaria was treated frequently in colonial health care facilities by the end of colonial rule in Ghana and Côte d’Ivoire (Fig 3), but the motive for the colonial response – at least initially – differed from yaws. Europeans had suffered severely or died from malaria in Africa since the 18^th^ century, prompting special attention of colonial administrations in the form of four key prevention strategies introduced in the early 20^th^ century: eradication of and protection against mosquitos carrying the disease, the use of prophylactic quinine in treatment, and a segregation policy for those who were afflicted [1,74]. The issue was especially urgent in West Africa, with a natural environment favourable to the main malaria vectors (the *Anopheles gambiae* and the *Anopheles funestus*), making the disease common in Ghana and Côte d’Ivoire by the 20^th^ century. With the introduction of quinine in the 19^th^ century, treatment was possible before formal colonial rule [75].

In Ghana, early colonial malaria policy largely focused on Europeans, although small-scale experiments with provision of preventive quinine and attempts at larval control were also taken. According to Patterson, many Ghanaians did not seek treatment from colonial health care facilities, contributing to the initially low number of cases treated per capita (Fig 3) [10]. In Côte d’Ivoire, racist colonial beliefs – suggesting that local populations did not suffer from malaria – contributed to the low official morbidity figures at the beginning of the 20^th^ century (Fig 3) [59]. Consequently, treatment was focused on Europeans, although to protect economic measures preventive measures were also extended to local labourers, which included preventive administration of quinine, destroying larvae and draining open water points, mosquito nets, and the provision of a substantial diet to local labourers [59].

Following general developments in the availability of colonial health care facilities and a broadening of the stance towards the provision of health care, the number of official malaria cases treated (per capita) started to increase in the two countries around 1930 (Fig 3). In Côte d’Ivoire, colonial administrators acknowledged the shifting trend, while the reports on Ghana fail to do so. The 1935 report on Côte d’Ivoire attributes the change to higher incidence among Europeans, and greater colonial health care uptake against the disease by the local population [26]. However, at the onset of WWII, malaria was deprioritised in colonial health care policy in Côte d’Ivoire in favour of other diseases, and the belief that mostly Europeans were affected was still present among colonial doctors [59]. Despite this stance in colonial policy, Fig 3 shows that the number of malaria cases treated per capita in Côte d’Ivoire continued to increase, and at a higher pace compared to Ghana as of the late 1930s. Several factors explain this difference: an increase in European population and a geographical widening of colonial malaria care in Côte d’Ivoire, paired with inadequate level of anti-malaria measures (such as insecticide DDT) due to a lack of resources and staff in Ghana during the 1940s and early 1950s [24]. By 1952, unsuccessful recruitment for a malariologist and drainage engineer – required for more elaborate anti-malaria measures – was seen as the major impediment to addressing malaria in Ghana [24]. The next year, investigations led by a medical officer of health and an entomologist started, which resulted in a report on the incidence of malaria in the colony that provided policy recommendations focused on large scale control measures (such as improving draining and the use of insecticides) [25]. By this time, malaria had become the principal cause of morbidity in Ghana.

### 4.2. Diseases prioritised for their effect on European populations

#### 4.2.1. *Sleeping sickness.*

Sleeping sickness is a key example of a disease that drew considerable attention from colonial authorities but prompted differing responses from the British and French. Endemic in parts of Africa – particularly in chronic form in West Africa – it gained widespread concern due to early 20^th^-century epidemics in East and Central Africa [6,45,46]. Lyons aptly describes sleeping sickness around the turn of the century as a ‘real social event’, attracting international medical researchers’ interest for its cause and colonial concerns for its effect on local labour supply [6]. Colonial administrations in Africa responded quickly to outbreaks of sleeping sickness for humanitarian, economic and scientific reasons.

Despite the disease’s longstanding endemic presence in Ghana, low official morbidity figures are reported (S2 and S3 Figs) [10]. Early 20^th^ century colonial investigations concluded that the tsetse fly was widespread, but that this did not translate into sleeping sickness epidemics [21,23]. Despite this conclusion, sleeping sickness was deemed a potential threat following investigations in Ashanti due to increased trade movements, and outbreaks elsewhere in Africa [46,21]. In the 1913 medical report, Principal Medical Officer Alexander claims: “The percentage of cases found infected out of the large number examined (110 in 39,742) would not appear to be by any means large, but it is sufficient to serve as a warning of its presence, in order that effective measures might be taken to prevent its spread” [21]. He proposes preventive policies through vector control and quarantine, before concluding with a warning: “The danger of the spread of this disease (owing to the easier means of communication that now exists) to districts where it has not previously been endemic and where it might become epidemic must not be forgotten” [21]. Notably, unlike malaria policy – often focused on protecting European populations – this suggests concern for the local population and appeared to have been motivating proposed measures of which the focus on vector control aligned with British tropical medicine’s growing focus on disease vectors [45].

By the 1930s, the fears materialized with the outbreak of a major epidemic in the Northern Territories (S3 Fig) [10,24]. The colonial medical reports initially attribute the increases in sleeping sickness cases to improved recordkeeping, to a larger focus of health care policy on the disease in the recent years, and to immigrants from French colonial territory [24]. Once recognized as an epidemic by colonial administrators, measures such as preventive bush clearing against the tsetse fly and providing treatment to infected persons – that had been introduced in the previous decades – were intensified [23,24].

In 1932, British and French authorities began a combined sleeping sickness treatment campaign, with mobile medical teams for Côte d’Ivoire operating in Ghana – an approach based on 1920s French campaigns in Central Africa [11,24,26]. In 1937, the British colonial administration launched a new organization to send out their own mobile medical teams [10]. The colonial policies of bush clearing and surveying and mass-treating the population turned out to be effective in Ghana: official morbidity figures declined in the 1940s (S3 Fig, also in absolute terms), and by the 1940s sleeping sickness was limited to small northern areas.

**Fig 4:**
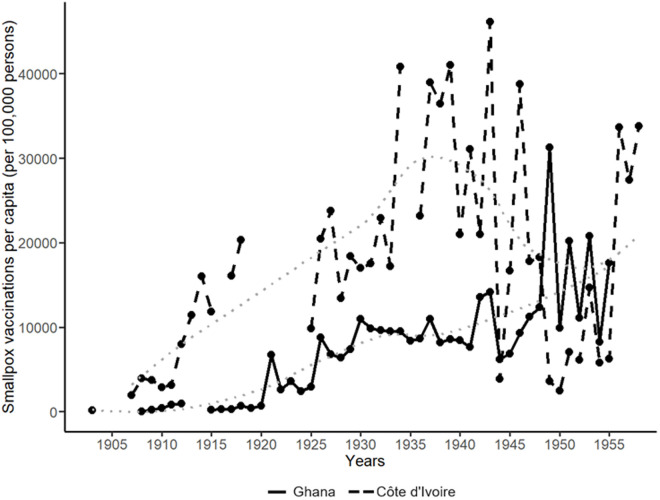
Smallpox vaccinations per capita (per 100,000 persons) administered by colonial smallpox campaigns in Ghana and Côte d’Ivoire, 1902-1957. Note: the dotted lines represent the (curved LOESS) trends for Ghana and Côte d’Ivoire. Data source: [14,20–30]. See S28–S29 Text for structural breaks tests.

In Côte d’Ivoire, the tsetse fly was widely present, and sleeping sickness became a growing concern by the early 1930s. The 1932 medical report highlights the threat, and official morbidity figures rose considerably after the inclusion of parts of Upper Volta to the colony in 1933 (S2 Fig) [26,76]. By 1937, it was considered the leading cause of overall morbidity (concentrated in the north and Volta regions), though yaws remained more frequently treated in health facilities (S2 Fig and Fig 4) [26].

Unlike Ghana, French colonial policy prioritized treatment methods over vector control in Côte d’Ivoire. Temporary nurses were recruited who received six months of specialist training in diagnosing sleeping sickness [26]. Standard treatment involved lumbar punctions, followed by treatment with the arsenic-based drug axtoxyl or orsanine, which caused severe side-effects including blindness and disease aggravation [11,46,26]. From the 1930s, colonial mobile medical teams expanded detection efforts, and in 1939 sleeping sickness was formally included in the new AOF service for several epidemic diseases [59,26]. The *Service General Autonome de la Maladie du Sommeil* continued to focus on detection and drug-based treatment of sleeping sickness, particularly with atoxyl and trypanocides.

#### 4.2.2. *Yellow fever.*

Yellow fever also stands out as a key concern in colonial health policy in Ghana and Côte d’Ivoire, despite relatively low official morbidity figures (S4 Fig). Its prominence was due to its historic toll on Europeans in West Africa – alongside malaria, it was a leading cause of deaths in the 18^th^ century – as well as recurring epidemics (18^th^ to 20^th^ century) and growing medical interest [1,77]. The cause of the disease was initially debated during the 19^th^ century, with possible explanations ranging from miasmas to poisons, until it was identified in 1900 as vector-borne through the *A. aegypti* mosquito [50,77]. Subsequently, preventive measures centred on vector control and, by the 1930s, vaccinations. The absence of effective treatment likely contributes to the low level of official morbidity figures (S4 Fig; S31 Text) [37].

In this context, British colonial policymakers in Ghana considered yellow fever a major threat due to its effect on the European population [10]. An outbreak in Sekondi and Accra in 1910–1911, considered to be an epidemic, shaped colonial health policy in the subsequent decades by prompting segregation policies, quarantines and vector control [21]. It consisted of ten cases of yellow fever in Sekondi among Europeans, of whom nine died, followed by three deaths from the local population and two other unspecified cases in 1910 [21]. Nine additional cases were reported for 1911, of which seven were found in Accra [21]. Despite these measures, and lacking an effective treatment, several other outbreaks of yellow fever in Ghana followed (S4 Fig), including a deadly epidemic in 1937 that claimed 69 out of 75 reported cases – mostly among the local population, a stark contrast to the previous epidemic [24]. The 1937 report suggests that ‘many more fatal cases must have occurred than came to official notice’ and only a small percentage of total cases was reported [24]. In response, colonial administrators maintained earlier measures while expanding sanitation education and inoculation, which became more common among the European population by the late 1930s [24]. In subsequent decades, large-scale colonial vaccination campaigns were introduced (S19 Table), which were not always carried out successfully. For example, the mass vaccination campaign in 1951 vaccinated over 330,000 persons in the area between Winneba and Akwapim, but was halted due to medical complications observed in Nigeria and supervision problems in Ghana [10].

In Côte d’Ivoire, severe yellow fever epidemics around 1900 resulted in the opening of a first infirmary for Europeans in 1902 [36]. Early French colonial policy focused on improving sanitation and water provision, and imposing quarantines (against outbreaks within Ghana and other British colonies) [26]. Sanitation measures against yellow fever were also implemented in other French colonies, such as St. Louis in Senegal. As Ngalamulume argues, yellow fever challenged the *mission civilisatrice* in the late 19th and early 20th centuries: widespread illness and death, without an effective colonial response, undermined the legitimacy of colonialism as a vehicle for spreading Western medicine [50]. In the subsequent decades, French colonial policy towards yellow fever continued to expand in Côte d’Ivoire. A vaccine was developed by 1932, and a 1933 local decree (adjusted in 1938) mandated vector control, vaccinations and installing fences as policy measures [37,26]. Initially, a small number of vaccines was administered (S19 Table), mostly to Europeans and Syrians [28]. A more extensive campaign in 1940, combined with smallpox vaccination, initiated a recurring campaign across French West Africa, with mass vaccinations roughly every four years (S19 Table) [78].

### 4.3. Diseases prioritized due to availability of preventive treatment

#### 4.3.1. *Smallpox.*

Even more so than other diseases, smallpox received considerable attention from colonial administrations [14]. It had a long presence in West Africa, predating European exploration in the 15^th^ century and the slave trades in the 16^th^ century [79]. Variolation was widely used as a preventive method in African societies prior to colonial rule. Despite its long history, relatively few cases (per capita) were treated in colonial health care facilities in Ghana and Côte d’Ivoire (S5 Fig).

Several outbreaks occurred in Ghana and Côte d’Ivoire between 1897 and 1957 (S5 Fig) [14,24], prompting elaborate colonial policy measures focused on tracking, isolating and preventing smallpox cases, as no effective curative treatment existed. Most importantly, Jenner’s vaccination method, long established in Europe, was introduced in African colonies [14]. The weight given to preventive policy in the two countries becomes clear from reviewing the colonial medical reports; mentions of (outbreaks of) smallpox tend to go hand in hand with notions of isolation measures, (the number of) vaccinations and/or other preventive measures (see, e.g., the colonial medical reports from 1900 and 1934 for Ghana, and 1905 and 1939 for Côte d’Ivoire) [21,24,26]. This focus of British and French colonial policymakers on prevention of smallpox through vaccinations, which occurred mostly outside of colonial health care facilities, paired with a lack of treatment options, also explains why the official morbidity figures were low in both countries (S5 Fig). The development of smallpox campaigns and other colonial vaccination campaigns in Ghana and Côte d’Ivoire is analysed in more detail in Subsection 4.5.

### 4.4. Beyond the priorities: colonial responses to other diseases

The remaining diseases in the novel dataset did not fit into either of the previous groups, but can be categorized by their observed distinct patterns depending on colonizer and a remnant category.

The first category of diseases in this subsection consists of leprosy, measles, dysentery, gonorrhoea and syphilis. Each of these diseases was commonly prevalent in both countries. Official morbidity figures (per capita) all start out low in both countries and subsequently rise in Côte d’Ivoire during the 1930s/1940s, while cases in Ghana lag behind (S6–S11 Figs) – motivating why these five diseases can be grouped together. In part, the timing of the increase in Côte d’Ivoire and the observed difference between the two countries can be explained by general developments including changes in population and in overall colonial policy, and the expansion of the colonial health care network over time (see S31 and S32 Texts for more detail). This subsection focuses on discussing and comparing the disease-specific reasons for this observed difference between Ghana and Côte d’Ivoire.

Regarding leprosy, a change in French colonial policy contributed to the observed difference between Côte d’Ivoire and Ghana in S6 Fig. A new approach to leprosy commenced in 1932 in Côte d’Ivoire. The French colonial medical service began screening patients for leprosy across the colony, and ‘segregation villages’ for lepers were to be built in all *cercles* [26]. Moreover, a central institute for leprosy was constructed in Mali (formerly French Sudan in AOF), that was tasked with researching the origin and spread of the disease, and possible methods for treatment and prevention [37]. Reported official morbidity figures in Côte d’Ivoire started to rise in the years after imposing the new policy (S6 Fig). Although the medical reports on Ghana discuss some policies relating to segregation of patients with leprosy, an extensive colonial programme like the French approach did not seem to have been imposed.

For measles, the epidemic nature of the disease contributes to the observed difference between the two countries. An outbreak of measles occurred in Côte d’Ivoire during 1939 and 1940, that translated into a higher number of official morbidity figures (S7 Fig). The 1939 colonial medical report recognizes this increase in cases, and describes that it was spread out across the colony, with major outbreaks in Ferkéssédougou, Ouagadougou and Bongouanou (amongst others) [26]. The majority out of the total cases (60.13%) was said to have been in children aged 0–5. It attributes the continued rise in 1940 to an increased number of afflicted adults (78.84% of total cases), resulting from the movement of soldiers to different garrisons and posts in the colony [28]. The disease again took on an epidemic form in 1944 (S7 Fig) and 1945, when Lahou and Gagnoa suffered from especially large outbreaks [59].

Contrary to this attention paid by the French medical reports, measles was generally overlooked by British colonial policymakers – despite its common occurrence in Ghana [10]. In addition, the local population tried to conceal measles cases to prevent the imposition of quarantine measures by the British administration. This lack of British colonial response can be illustrated by the apparent measles outbreak of 1952 in Ghana, based on official morbidity figures (potentially sustaining into 1953, albeit at a relatively lower level; S7 Fig). This development is not recognized by British colonial administrators, instead stating that ‘there has been an absence of epidemics in the larger municipalities’ [25].

Dysentery was commonly prevalent in both countries during colonial rule [10,26]. In Ghana, treatment methods were available for the amoebic variant and the bacillary variant (as of the 1930s when sulpha was introduced), but they reached few official patients (S8 Fig) [10]. British policymakers considered poor sanitation and a lack of modern sewage disposals as the root cause of the high incidence of dysentery in the colony [25]. However, no specific policy measures seem to have been implemented against the disease by the British colonial administration. In contrast, S8 Fig shows that official morbidity figures start to rise around the 1930s. Three factors contribute to this divergence. Firstly, dysentery started to take on epidemic proportions in Côte d’Ivoire during the 1930s, especially in urban centres [26]. The inclusion of Haute-Volta in 1933 to Côte d’Ivoire also led to a rise in patient figures [26]. Moreover, Rouanet describes how French colonial health policy was implemented that surveyed and treated the population for dysentery (with emetine and Stovarsol) [37].

Gonorrhoea and syphilis were the predominant venereal diseases in both colonies and occurred frequently [61,26]. The 1938 report on Côte d’Ivoire attributes the rapid increase in official venereal disease cases during the 1930s to increased movement of people – causing the diseases to spread from urban to rural locations (S9 and S10 Figs) [28]. Regulation of prostitution, as decreed on April 18, 1940, was deemed the only policy measure available to combat the diseases [28]. Official morbidity figures do not reflect the common occurrence of these venereal diseases in Ghana (S9–S11 Figs), even as gonorrhoea and syphilis became more widespread following increased mobility and social change [10]. As discussed more extensively in S31 Text, explanations for this difference include a reliance on other providers of health care (traditional medicine and missionary care), a lack of treatment methods for most of the period, and a possible reluctance of British administrators to intervene in sexual practices – similar to Vaughan’s observations for Uganda [13].

In sum, it becomes apparent that the formal British and French colonial policy shifts towards social development mid-20^th^ century can be related to official numbers of cases treated for these five diseases in Côte d’Ivoire, whereas it cannot be found for Ghana. Differences in how morbidity trends were acknowledged and addressed underscore this contrast. From the 1930s onward, French colonial health policy increasingly recognized the significance of diseases like leprosy, measles, dysentery, gonorrhoea, and syphilis to population health. British colonial policy, by contrast, failed to respond to these threats with the same urgency or consistency. Similar conclusions have been drawn by Patterson and Bannister regarding river blindness in Northern Ghana [47,48]. Overall, the trajectory of this disease group illustrates how colonial responses in Ghana and Côte d’Ivoire were shaped not only by factors discussed previously – such as incidence, European exposure, and treatment availability – but also by key differences in administrative responsiveness.

The remaining diseases – chickenpox, beriberi, influenza, and tuberculosis – do not neatly fit into the earlier categories, nor do they share clear trends or patterns. This subsection concludes the analysis of the colonial response to official morbidity developments by briefly addressing each disease individually. A more detailed discussion of S12–S15 Figs is available in S31 Text.

The British and French colonial response to chickenpox was influenced by its clinical similarity to smallpox (see S30 Text). In Côte d’Ivoire, the 1938 medical report noted that nurses and auxiliary doctors often confused the two when faced with severe complications [28]. French administrators conducted health surveys on chickenpox during the early 20th century [37], but British policymakers showed little concern [10]. Its similarity to smallpox may explain the rising morbidity trends shown in S12 Fig, as chickenpox cases were increasingly uncovered through colonial smallpox vaccination programmes.

Despite being endemic, official morbidity figures for beriberi were relatively low in both countries (S12 Fig) [27,80]. Although British colonial medicine researchers studied its causes, this interest was not reflected in Ghana’s official morbidity figures in Ghana (S13 Fig) [80–82]. No meaningful colonial health policies were implemented until the late colonial period. French colonial policymakers began linking nutrition to labour productivity around WWI, but failed to implement effective interventions [83]. The 1949 Conference on Food and Nutrition in Africa, attended by British and French colonial policymakers and members of the WHO and FAO, marked a turning point by recognizing malnutrition in Africa as a public health issue and creating urgency among international organizations to focus on providing aid in the 1950s [37].

Influenza stands out not for its routine treatment or policy attention but for the exceptional 1918 pandemic, caused by a more virulent variant of the virus, and can clearly be observed for Ghana in S14 Fig (and to a lesser extent in S25–S27 Tables) [84]. The disease first arrived in Cape Coast on August 31, 1918, via infected passengers of the S.S. Shonga, spreading rapidly across the colony despite quarantine and treatment methods between September 1918 and January 1919 [10,21,85]. By 1919, an estimate of up to 100,000 deaths could be attributed to the disease. No colonial medical reports are available for Côte d’Ivoire from 1918 to 1920, but secondary sources suggest that the disease first arrived in Bassam on September 29, and spread similarly, with some calling it the worst epidemic in the history of Côte d’Ivoire [59,86]. The pandemic highlights the underdevelopment of both British and French colonial health care provision in the early 20^th^ century. Due to their narrowly focused health policies, British and French colonial policymakers were ill-equipped to handle the influenza pandemic. Although the policy expansion starting in the 1920s came too late to prevent its impact, it could have offered a stronger foundation for public health responses.

Tuberculosis is one of the few diseases showing a clear upwards trend in Ghana’s official morbidity figures since the early decades of colonial rule, especially during the 1920s (S15 Fig). The 1925 colonial medical report links this to both a rising incidence and a growing willingness to be treated by the local population [23]. Nevertheless, apart from appointing a tuberculosis officer for Ghana in 1929, colonial policy measures remained minimal until 1935 [23]. By then, tuberculosis was ‘believed to constitute one of the greatest menaces to the indigenous population’ [24]. This shift in British colonial view was more widespread in West Africa in the 1930s, resulting in new preventive policies in Ghana focused on sanitation, urban planning and sanitary education [61,24].

In Côte d’Ivoire, the early decades of the 20^th^ century are marked by French colonial inaction in response to the development of official morbidity figures for tuberculosis (S15 Fig). A shift occurred in the 1920s with the introduction of BCG vaccination programmes, initially occurring in maternity health care facilities in AOF (1924), followed by vaccinations of children in urban areas (1927) [59]. As detailed in the next subsection, extensive vaccination campaigns were implemented towards the end of colonial rule (S19–S23 Tables).

### 4.5. Colonial vaccination programmes in Ghana and Côte d’Ivoire

In response to the development of official morbidity figures during the 19^th^ and 20^th^ century, colonial administrations in Africa introduced various prevention and control methods, including vaccination campaigns. Before 20^th^ century colonial rule, smallpox vaccinations had been successfully administered for decades in European countries [14]. As a result, smallpox became one of the earliest and most widespread targets of colonial campaigns. In addition to smallpox, campaigns intended to prevent and treat diseases such as yaws, syphilis, and yellow fever were also introduced in various African colonies [51].

Based on this article’s new dataset of official vaccination figures, Fig 4, S16 Fig, and S19 Table show that multiple colonial vaccination campaigns were introduced Ghana and Côte d’Ivoire. In Ghana, inoculations against the plague were performed in 1908, and mixed vaccines for yellow fever and smallpox were administered in 1952 (S19 Table). Aside from these, the medical reports on Ghana show only relatively substantial smallpox vaccination campaigns. In Côte d’Ivoire, a wider range of vaccinations was recorded from the 1930s, including those against malaria and yellow fever (S16–S17 Figs and S19 Table). Tuberculosis vaccinations with BCG, introduced in 1949, stand out for targeting schoolchildren and maternity patients rather than the general population [28]. As in Ghana, smallpox vaccinations outnumbered all others, although mixed vaccines and BCG also featured prominently (Fig 4, S16 Fig, S19 Table). 

Qualitative descriptions in colonial medial reports reinforce the perceived importance of smallpox campaigns. In 1910, first class Medical Officer of the colonial troops Guerchet describes how he ‘first dealt with the very important question of the vaccine’ while stationed in Bingerville, and a 1912 report credited the absence of major epidemics to prior vaccination campaigns [26]. The 1931 Gold Coast Medical Report considers vaccinations ‘the keystone of preventive measures’, and the 1932 report linked reduced case numbers to increased vaccinations, especially for the Eastern Province [24]. Given the size of smallpox vaccination campaigns and their prominence in policy considerations, the remainder of this subsection focuses on their development in more detail.

Fig 4 shows that smallpox vaccination campaigns started much earlier in both countries than previously suggested by Schneider [14]. In 1902, 4624 ‘successful’ vaccinations are reported in Ghana, compared to 28599 vaccinations in Côte d’Ivoire in 1906 (respectively 1.62 versus 19.63 smallpox vaccinations per 1,000 persons in per capita terms; Fig 4). By the late 1910s, the French colonial smallpox vaccination programme in Côte d’Ivoire was already quite extensive, whereas British campaigns remained comparatively limited (Fig 4).

Three factors explain this early divergence. Firstly, a smallpox epidemic occurred in 1905–1906 in Côte d’Ivoire, ‘raging without respite’ in nearly all regions, prompting French colonial administrators to send available doctors on vaccination campaigns [27]. No comparable epidemic is reported for Ghana, and Principal Medical Officer Henderson cites accounting issues in the 1902 report and claims that the reported figure potentially underestimates the total number of vaccinations as a result [21]. Finally, the enforcement of British and French colonial health policy differed: mandatory smallpox vaccinations were required by AOF law in French West Africa as of 1904, and systemic vaccinations started by 1905 (Fig 4) [14]. In Ghana, smallpox vaccinations only became compulsory for adults with the 1920 Vaccination Ordinance, extended to children in 1929 [24].

For the overall period, the comparative analysis also finds higher vaccination rates in Côte d’Ivoire than in Ghana across nearly all years (Fig 4) [14]. In addition to the previous contributing factors, the type of vaccine used by the two colonial administrations also differed, as did their production and transport, and their effectivity and availability.

In Côte d’Ivoire, local production of smallpox vaccines started in the late 19^th^ century. A production facility in Senegal provided smallpox vaccines to the French West African region in 1895, and the Pasteur Institute in Guinea began production in 1905 [14]. Reporting a lack of means and staff for vaccination efforts during the 1905 smallpox epidemic, French colonial administrators decided to move the production of vaccines to Côte d’Ivoire itself by 1916, producing nearly 400,000 doses in its first year [26,27]. By 1928, the use of vaccines from Bouaké allowed for over 1,500,000 inoculations with lymph [26]. Afterwards, dried vaccines became available, that were more stable in long distance transport, and the production of fresh vaccines moved from Bouaké to Upper Volta [26]. Despite the more elaborate infrastructure, French colonial reports also note staff shortages and local resistance against vaccination, both limiting vaccination campaigns until at least the 1930s [26].

In contrast, medical reports on Ghana suggest that British campaigns relied on lymph-based vaccines from the Lister institute in England by 1938, though some production tests were conducted in Nigeria [24]. These vaccines were more unstable, losing potency during the transport from Britain. By 1952, mixed smallpox and yellow fever vaccines used in Ghana were produced at the Pasteur Institute in Dakar [25]. Other factors contributing to the difference observed in Fig 4 include a shortage of vaccinators, a lack of available lymph throughout the period, and a declining success ratio of administered vaccinations (S17 Fig) [21,24]. Although no success ratios are available for Côte d’Ivoire, the 1931 medical report lists ‘positive’ percentages of smallpox vaccinations for the lymph vaccines from Bouaké (S20 Table) [26]. These percentages seem to suggest that the success ratio of smallpox vaccines in Côte d’Ivoire is indeed higher than the success ratio in Ghana.

### 4.6. Synthesis of the comparative analysis

This subsection draws together the main findings in a comparative perspective. From the analysis of official morbidity figures (Sections 4.1–4.4) and colonial vaccination campaigns (Section 4.5), several patterns emerge that highlight both similarities and differences in British and French colonial health responses in Ghana and Côte d’Ivoire. Firstly, the findings suggest that colonial responses were not solely driven by morbidity within colonial health care facilities. Although yaws and malaria were frequently treated in both countries, other diseases – such as yellow fever and smallpox – received considerable attention from the colonial administrations despite comparatively low official figures. These cases reflect how colonial concerns were often drive by the perceived threat of a disease to European settlers, economic objectives, or the availability of the preventive treatment, rather than by the disease burden in the local population.

Secondly, notable differences can be observed in how the British and French colonial administrations responded to disease trends, despite facing broadly similar (medical) developments (S32). One possible explanation for these differences lies in broader differences in colonial policies. While the literature has pointed to the distinction between direct and indirect rule as shaping state-building outcomes in former colonized countries, these institutional approaches may also have influenced the design and implementation of public health interventions. The results of this article suggest that the French administration in Côte d’Ivoire responded more frequently to shifts in official morbidity by expanding medical campaigns or adapting treatment strategies (e.g., for leprosy, venereal disease, and dysentery), whereas similar patterns are less consistently observed for Ghana. In this respect, the French colonial health response could be characterised as more centrally directed and policy-driven, especially from 1930s onward, in line with broader policy shifts in AOF. By contrast, the British approach in Ghana often appeared more fragmented or reactive based on the colonial medical reports, particularly for diseases not perceived as posing a direct threat to economic outcomes or European health.

Thirdly, the difference in colonial response also points to the relevance of institutional capacity and administrative prioritization. While both colonial governments expanded their health care provision over time, the scale and intensity of medical campaigns are shown to have differed [35]. French colonial vaccination campaigns are suggested to be broader in scope and quantity. However, it should be emphasized that data limitations make it difficult to fully evaluate the reach and effectiveness of these programmes (Section 2).

Finally, it is important to reiterate that the results in this article are based on official figures from colonial health care facilities and vaccination campaigns. As such, the scope of the article reflects only part of the overall health care provision in Ghana and Côte d’Ivoire. The limited availability of quantitative information on traditional medicine, missionary health care, and the local population’s strategies of resistance or accommodations in the records imposes interpretive constraints, and the exclusion of these aspects is not intended to disregard their importance. Nonetheless, the comparative analysis presented here suggests that while shared factors such as the expansion of colonial health infrastructure and changes in Western medicine shaped colonial health policy in both countries, the nature and timing of colonial responses were also mediated by institutional structures and administrative practices that varied between British and French rule.

## 5. Discussion and conclusions

This article analysed the British and French colonial response to the development of official morbidity figures for selected diseases in Ghana and Côte d’Ivoire during the first half of the 20^th^ century by combining new quantitative evidence from colonial records with a contextualised analysis of colonial policy and interventions The approach built on a theoretical framework of existing literature on European involvement in African disease environments and health care and policy under colonial rule, and follows the tradition in economic history of embedding analytical perspectives within historical interpretation. By combining archival sources, newly constructed datasets, and secondary literature, the article offers a comparative lens on how morbidity patterns were recognised and acted upon in the two neighbouring countries with differing colonial histories.

The results in Section 4 provide evidence that in the case of yaws and malaria, British and French colonial administrations responded to the relatively high number of patients treated in colonial health care facilities in the two countries. Three other diseases (sleeping sickness, yellow fever, and smallpox) were shown to be deemed important by British and French colonial policymakers for reasons other than their hospital incidence, and thus warranted a colonial response. Sleeping sickness and yellow fever were discussed to have received colonial attention because of their influence on the European population, while the potential to implement an existing preventive policy (vaccines) motivated the colonial focus on smallpox. Despite these similar influences, Section 4 also showed that British and French policy measures did not necessarily develop along the same lines. The British and French approach to yellow fever in Ghana and Côte d’Ivoire was found to be alike, prompted by a lack of alternative approaches and by akin public health movements in Great Britain and France. In contrast, sleeping sickness was met with different colonial responses in the two countries. The British approach emphasizing vector control, whereas the French turned more towards treatment methods.

The remainder of the selected diseases were discussed in Subsection 4.4. Five of these diseases (leprosy, dysentery, measles, gonorrhoea and syphilis) were commonly prevalent in Ghana and Côte d’Ivoire. Their development in official morbidity figure (per capita) was found to display a similar trend: after a relatively low number of cases (per capita) in both countries prior to the 1930s, the trend stays comparatively constant for Ghana while cases (per capita) in Côte d’Ivoire start to rise. It is argued that this divergence can be attributed to a difference in the French and British response, as the British failed to sufficiently recognize the importance of the influence of these five diseases on the local population’s health in Ghana. Colonial policymakers in Côte d’Ivoire seemed to become more aware once French colonial health policy broadened its general view after the initial decades of the 20^th^ century.

This article also studied the development of colonial vaccination programmes, and in particular smallpox vaccinations, because of their importance as part of the colonial response outside colonial health care facilities. Subsection 4.5 showed that French colonial policymakers in Côte d’Ivoire targeted a wider variety of diseases compared to the British administration in Ghana, even if smallpox vaccination campaigns trumped all other vaccination campaigns in the two countries. The subsequent analysis on colonial vaccination campaigns is the first to quantify the early smallpox vaccination programmes (prior to the mid-1920s) in Ghana and Côte d’Ivoire in particular, using new data from colonial sources, which forms a significant contribution to the previous literature. Subsection 4.5 also contributes to the seminal work by Schneider by using a different methodology, that allows for a comparative analysis between British and French policy by using per capita figures [14]. The results in this subsection provide evidence that a clear difference in colonial smallpox vaccination campaigns can be established between British and French health policy in the two countries. It finds that the number of smallpox vaccines administered per capita was higher in Côte d’Ivoire than in Ghana for nearly all years, which is argued to be explained by variations in colonial approaches. In particular, British mandatory smallpox vaccinations were introduced later in Ghana, and the type of vaccine used by French colonial health care provision in Côte d’Ivoire was more stable and produced locally. The synthesis of the findings (Section 4.6) underscores how similarities in disease environments and medical advances were accompanied by differences in colonial responses in Ghana and Côte d’Ivoire.

Although West Africa was historically deemed the ‘white man’s grave’ and a prominent breeding ground for many diseases, the findings of this article indicate that the number of cases treated (per capita) in colonial health care facilities in Ghana and Côte d’Ivoire did not necessarily match this widespread incidence of diseases. The colonial response to developments in official morbidity figures was influenced by an interconnected framework of the perceived prevalence of diseases, factors warranting immediate attention (including the effect on the European population and availability of treatment methods), the number of patients entering colonial health care facilities, and budgetary and personnel policy considerations. Vaccination campaigns formed an important aspect of preventive colonial health care policy in both countries, but greatly emphasized the threat of smallpox. Overall, colonial health care policies in Ghana and Côte d’Ivoire did not operate in isolation, but were influenced by colonial fiscal policy and capacity, the availability and effectivity of preventive methods and treatment, trust in medicine and other general developments. In this context, differing administrative strategies may have shaped the contrasting responses observed, underscoring the relevance of institutional frameworks in examining public health policy. This article contributed to the literature by making a first step in disentangling this complex relationship between (changes in) the disease environment during colonial rule in Africa, its effect on admissions in colonial health care facilities, and the policy response by different colonial rulers. As for the present, the findings in this article suggest that policymakers in Ghana and Côte d’Ivoire today should consider past developments in disease patterns and distribution, including but not limited to those during colonial rule, when devising new policy. The differences shown between historical disease patterns, between forces influencing policy decision-making, and between the British and French colonial administrations, underscore the importance of considering local conditions and local disease burdens in designing present-day public health care policies.

## Supporting information

S1 Fig Jaundice cases in colonial health care facilities per 10,000 persons, ca.1900–1940: Ghana and Côte d’Ivoire. (PDF)

S2 Fig Sleeping sickness cases in colonial health care facilities per 10,000 persons, ca.1900–1955: Ghana and Côte d’Ivoire. (PDF)

S3 Fig Sleeping sickness cases in colonial health care facilities in Ghana per 10,000 persons, ca.1900–1955. (PDF)

S4 Fig Yellow fever cases in colonial health care facilities per 10,000 persons, ca.1900–1955: Ghana and Côte d’Ivoire. (PDF)

S5 Fig Smallpox cases in colonial health care facilities per 10,000 persons, ca.1900–1955: Ghana and Côte d’Ivoire. (PDF)

S6 Fig Leprosy cases in colonial health care facilities per 10,000 persons, ca.1900–1955: Ghana and Côte d’Ivoire. (PDF)

S7 Fig Measles cases in colonial health care facilities per 10,000 persons, ca.1900–1955: Ghana and Côte d’Ivoire. (PDF)

S8 Fig Dysentery cases in colonial health care facilities per 10,000 persons, ca.1900–1955: Ghana and Côte d’Ivoire. (PDF)

S9 Fig Gonorrhoea cases in colonial health care facilities per 10,000 persons, ca.1900–1955: Ghana and Côte d’Ivoire. (PDF)

S10 Fig Syphilis cases in colonial health care facilities per 10,000 persons, ca.1900–1955: Ghana and Côte d’Ivoire. (PDF)

S11 Fig Syphilis cases in colonial health care facilities per 10,000 persons in Ghana, ca.1900–1955. (PDF)

S12 Fig Chickenpox cases in colonial health care facilities per 10,000 persons, ca.1900–1955: Ghana and Côte d’Ivoire. (PDF)

S13 Fig Beriberi cases in colonial health care facilities per 10,000 persons, ca.1900–1955: Ghana and Côte d’Ivoire. (PDF)

S14 Fig Influenza cases in colonial health care facilities per 10,000 persons, ca.1900–1955: Ghana and Côte d’Ivoire. (PDF)

S15 Fig Tuberculosis cases in colonial health care facilities per 10,000 persons, ca.1900–1955: Ghana and Côte d’Ivoire. (PDF)

S16 Fig Mixed yellow fever and smallpox vaccinations in Côte d’Ivoire (per 10,000 persons). (PDF)

S17 Fig Successful smallpox vaccinations as a percentage of the total number of smallpox vaccinations in Ghana. (PDF)

S18 Table Development of total number of colonial health care facilities and total number of patients treated (in colonial health care facilities), 1905–1952. (PDF)

S19 Table Colonial vaccinations in Ghana and Côte d’Ivoire against diseases other than smallpox. (PDF)

S20 Table Percentage of positive smallpox vaccinations in Côte d’Ivoire, 1925–1931. (PDF)

S21 Table Testing for structural breaks in smallpox vaccinations per capita. (PDF)

S22 Table Sensitivity checks structural break tests, smallpox vaccinations per capita: substituting missing values with last available year. (PDF)

S23 Table Sensitivity checks structural break tests, smallpox vaccinations per capita: using linear interpolation for missing values. (PDF)

S24 Text Data Appendix. (PDF)

S25 Table Côte d’Ivoire: total number of deaths per disease as a percentage of total cases per disease (beriberi – leprosy, rounded to two decimals). (PDF)

S26 Table Côte d’Ivoire: total number of deaths per disease as a percentage of total cases per disease (malaria – yellow fever, rounded to two decimals). (PDF)

S27 Table Ghana: total number of deaths per disease as a percentage of total cases per disease (beriberi – leprosy, rounded to two decimals). (PDF)

S28 Table Ghana: total number of deaths per disease as a percentage of total cases per disease (malaria – yellow fever, rounded to two decimals). (PDF)

S29 Text Structural breaks testing: smallpox vaccinations per capita. (PDF)

S30 Text Glossary of diseases. (PDF)

S31 Text Extended discussion of graphs. (PDF)

S32 Text Changing determinants of disease patterns and trends, and other dilemma’s. (PDF)

S33 Text Additional references in supporting information. (PDF)

S34 File Minimal data set. (XLSX)
