# Supplementary material for: The colonial response to the development of disease in Ghana and Côte d’Ivoire (ca. 1900-1955): A comparative analysis of British and French colonial health policies
Source: PLoS One. 2025 Aug 14;20(8):e0329713. doi: 10.1371/journal.pone.0329713 (PMC12352650; doi:10.1371/journal.pone.0329713)
Supplement: S1 Fig — (PDF) [file pone.0329713.s001.pdf]

S1 Fig. Jaundice cases in colonial health care facilities per 10,000 persons, ca. 1900-1940: Ghana and Côte d'Ivoire.

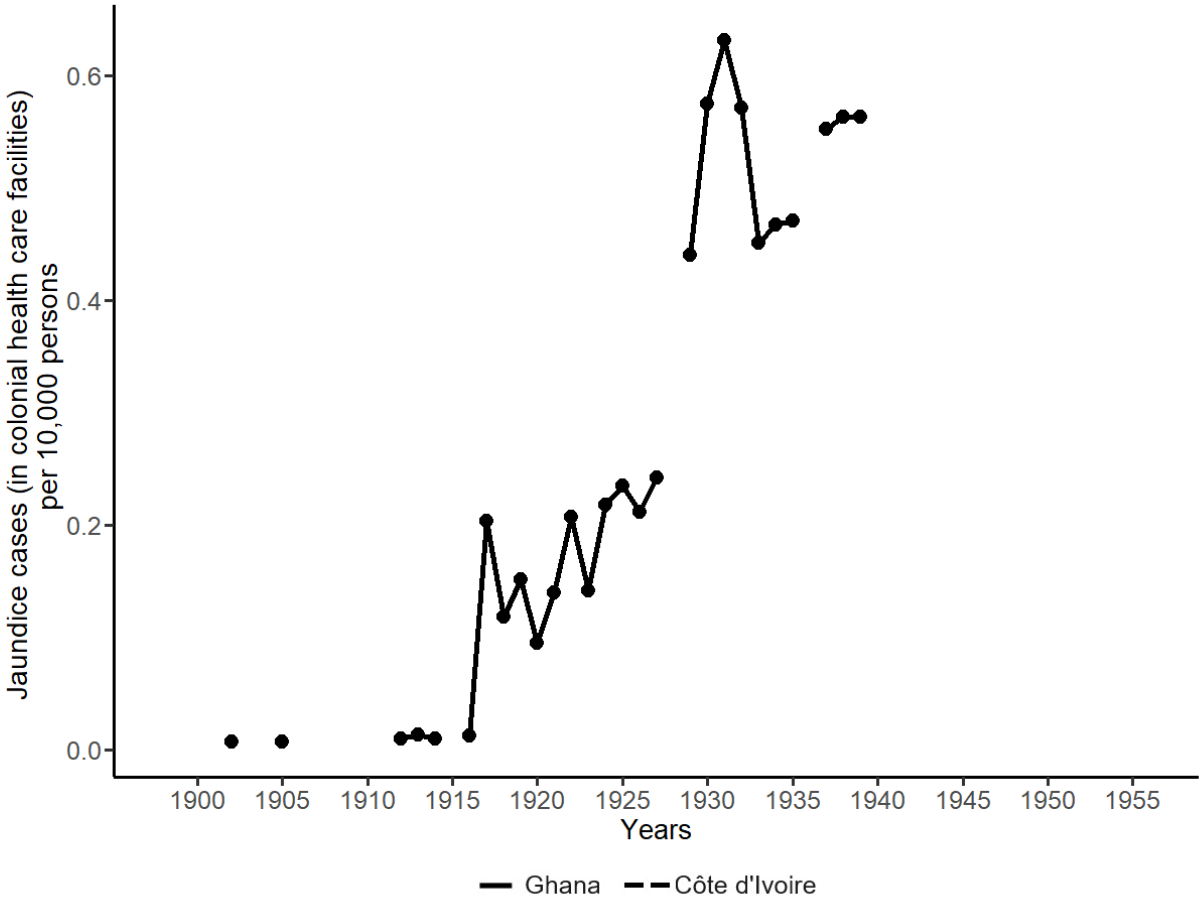

Data source: [52-55].
