# Supplementary material for: The colonial response to the development of disease in Ghana and Côte d’Ivoire (ca. 1900-1955): A comparative analysis of British and French colonial health policies
Source: PLoS One. 2025 Aug 14;20(8):e0329713. doi: 10.1371/journal.pone.0329713 (PMC12352650; doi:10.1371/journal.pone.0329713)
Supplement: S5 Fig — (PDF) [file pone.0329713.s005.pdf]

S5 Fig. Smallpox cases in colonial health care facilities per 10,000 persons, ca. 1900-1955: Ghana and Côte d'Ivoire. Note: the dotted lines represent the (curved LOESS) trends for Ghana and Côte d'Ivoire. The trendline for Côte d'Ivoire is composed of two parts: a linear trendline before the large increase, and a default LOESS smoothing afterwards. Using the LOESS method (or any other alternative) on the full timeseries results in a partially negative trendline, which has no meaningful interpretation given the data used. This note applies to all other figures in which a trend line composed of two parts is presented.

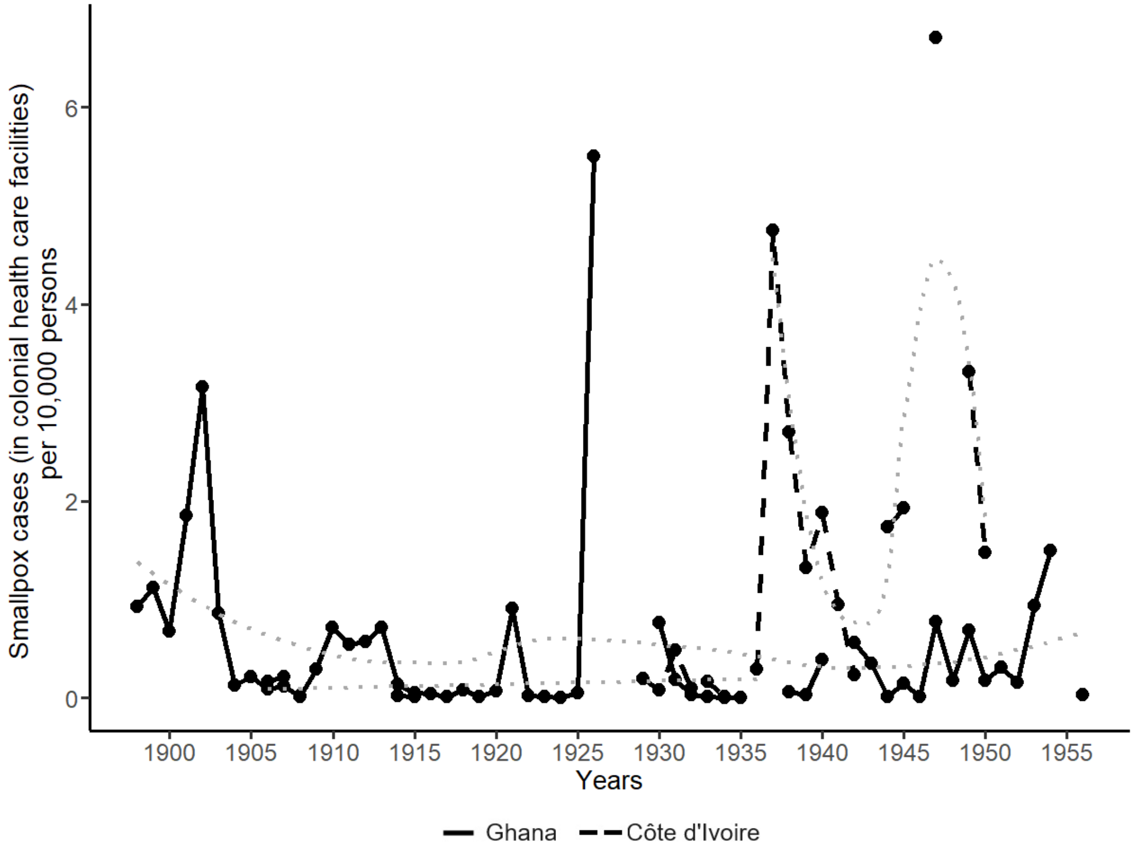

Data source: [52-60].
