# Supplementary figures and images for: The colonial response to the development of disease in Ghana and Côte d’Ivoire (ca. 1900-1955): A comparative analysis of British and French colonial health policies
Source: PLoS One. 2025 Aug 14;20(8):e0329713. doi: 10.1371/journal.pone.0329713 (PMC12352650; doi:10.1371/journal.pone.0329713)

**S11 Fig. Syphilis cases in colonial health care facilities per 10,000 persons in Ghana, ca. 1900-1955.**

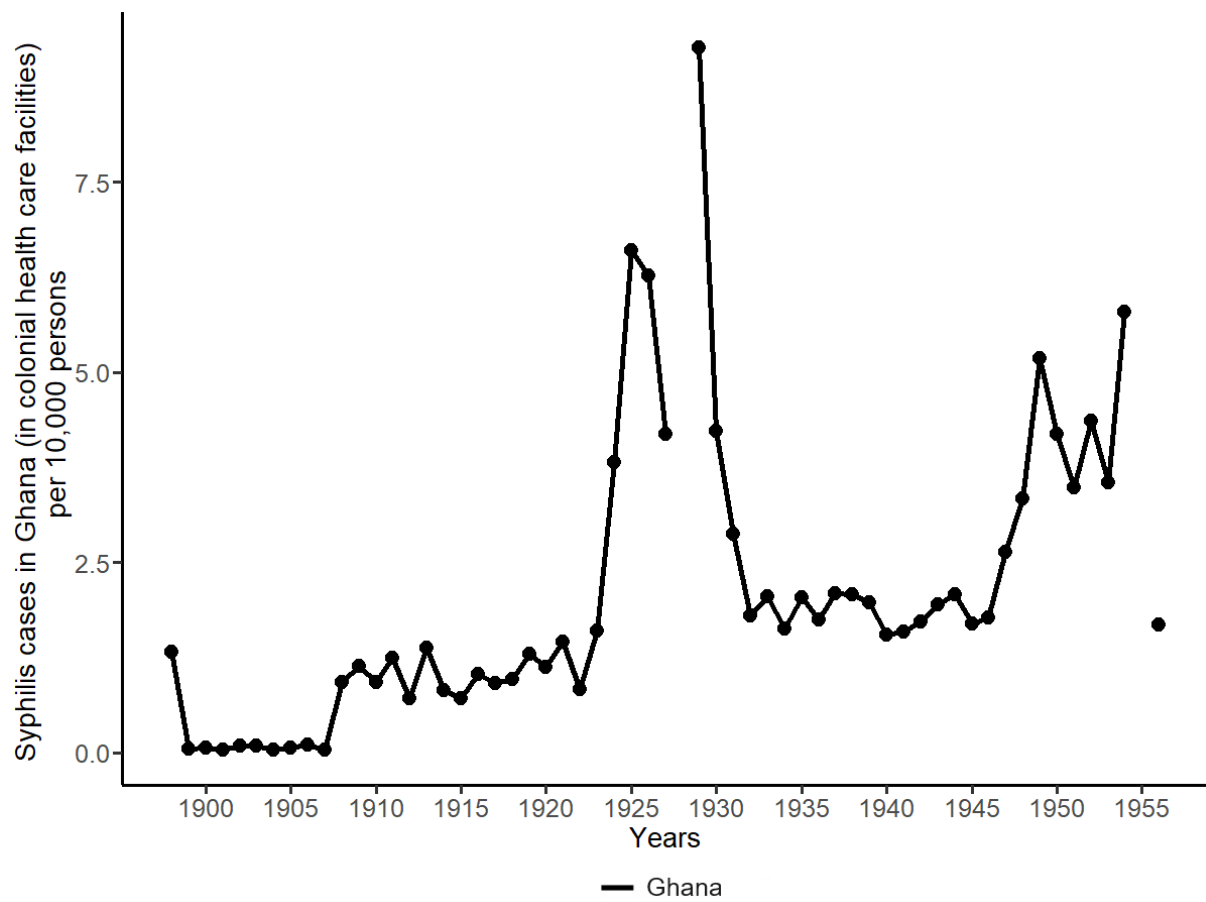

Data source: [52-56].

Supplement: S11 Fig — (PDF) [file pone.0329713.s011.pdf]
