# Supplementary material for: The colonial response to the development of disease in Ghana and Côte d’Ivoire (ca. 1900-1955): A comparative analysis of British and French colonial health policies
Source: PLoS One. 2025 Aug 14;20(8):e0329713. doi: 10.1371/journal.pone.0329713 (PMC12352650; doi:10.1371/journal.pone.0329713)
Supplement: S17 Fig — (PDF) [file pone.0329713.s017.pdf]

S17 Fig. Successful smallpox vaccinations as a percentage of the total number of smallpox vaccinations in Ghana. Note: the dotted line represents the (curved LOESS) trend.

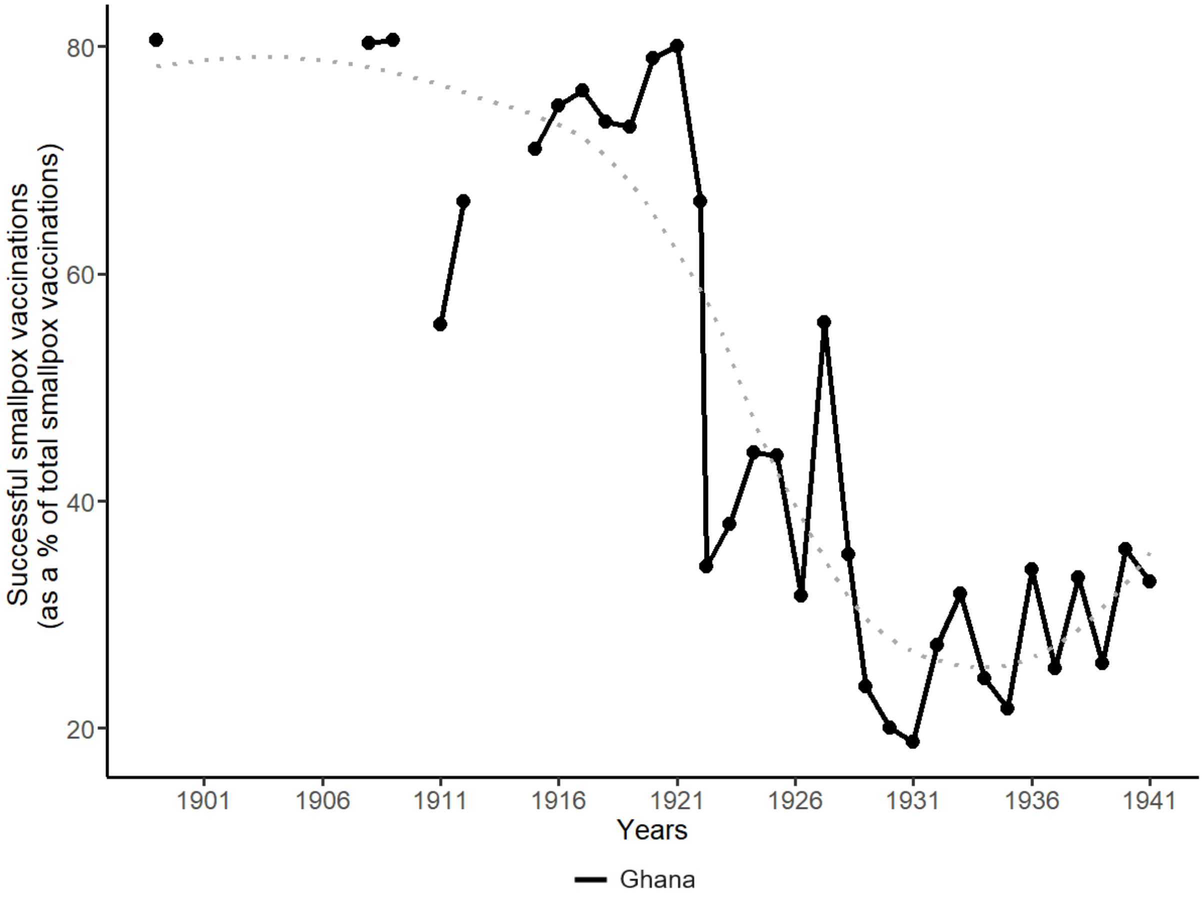

Data source: [52-55].
