# Supplementary material for: The colonial response to the development of disease in Ghana and Côte d’Ivoire (ca. 1900-1955): A comparative analysis of British and French colonial health policies
Source: PLoS One. 2025 Aug 14;20(8):e0329713. doi: 10.1371/journal.pone.0329713 (PMC12352650; doi:10.1371/journal.pone.0329713)
Supplement: S18 Table — (PDF) [file pone.0329713.s018.pdf]

S18 Table. Development of total number of colonial health care facilities and total number of patients treated (in colonial health care facilities), 1905-1952. Per 100,000 persons in parentheses, rounded to two decimals.

Year	Country	Colonial health care facilities	Patients treated
**Early 1900s**	**Ghana**	11 (0.38)	15,985 (546.42)
	**Côte d'Ivoire**	10 (0.70)	.
**1931**	**Ghana**	86 (2.22)	259,067 (6693.47)
	**Côte d'Ivoire**	53 (2.87)	30,955 (1673.95)
**Late colonial rule**	**Ghana**	57 (1.02)	1,038,787 (18633.46)
	**Côte d'Ivoire**	66 (3.15)	1,271,483 (60670.92)

Data source: [52-59, 87].
