# Supplementary material for: The colonial response to the development of disease in Ghana and Côte d’Ivoire (ca. 1900-1955): A comparative analysis of British and French colonial health policies
Source: PLoS One. 2025 Aug 14;20(8):e0329713. doi: 10.1371/journal.pone.0329713 (PMC12352650; doi:10.1371/journal.pone.0329713)
Supplement: S19 Table — (PDF) [file pone.0329713.s019.pdf]

S19 Table. Colonial vaccinations in Ghana and Côte d'Ivoire against diseases other than smallpox. Per capita figures (per 10,000 persons) in parentheses, rounded to two decimals.

**Year**	**Country**	**Disease**	**Total no. of vaccinations (per capita)**
**1908**	**Ghana**	Plague	34136 (inoculations) (113.66)
**1935**	**Côte d'Ivoire**	Malaria	2590 (13.25)
**1936**	**Côte d'Ivoire**	Malaria	326 (1.64)
		Yellow fever	7417 (37.42)
		Pneumococci	140 (0.71)
		Tetanus	58 (0.29)
**1937**	**Côte d'Ivoire**	Plague	7 (0.03)
		Cholera	7 (0.03)
		Malaria	140 (0.70)
		Meningococci	15 (0.07)
		Pneumococci	16 (0.08)
		Tetanus	13 (0.06)
		Gangrene	1 (0.00)
		Rabies	8 (0.04)
		Syphilis	150 (0.75)
**1938**	**Côte d'Ivoire**	Plague	24 (0.12)
		Malaria	351 (1.72)
		Yellow fever	351 (1.72)
		Meningococci	4 (0.02)
		Pneumococci	280 (1.37)

		Tetanus	14 (0.07)
		Gangrene	4 (0.02)
		Dysentery	4 (0.02)
		Rabies	48 (0.24)
		Syphilis	26 (0.13)
**1939**	**Côte d'Ivoire**	Malaria	613 (2.97)
		Yellow fever	603 (2.92)
		Meningococci	434850 (2104.01)
		Pneumococci	256 (1.24)
		Tetanus	151 (0.73)
		Typhus	1500 (7.26)
		Gangrene	643 (3.11)
		Dysentery	37 (0.18)
		Rabies	75 (0.36)
		Tuberculosis	42 (0.20)
		Syphilis	46 (0.22)
**1940**	**Côte d'Ivoire**	Meningococci	29238 (138.51)
		Pneumococci	11 (0.05)
		Diphtheria	89 (0.42)
		Tetanus	63 (0.30)
		Gangrene	56 (0.27)
		Dysentery	14 (0.27)
		Syphilis	34 (0.16)
		Rabies	80 (0.38)

**1941**	**Côte d'Ivoire**	Yellow fever	182888 (857.25)
		Meningococci	50709 (237.69)
		Pneumococci	31 (0.15)
		Diphtheria	7 (0.03)
		Tetanus	22 (0.10)
		Dysentery	1 (0.00)
		Rabies	500 (2.34)
**1943**	**Côte d'Ivoire**	Yellow fever	63800 (288.57)
		Tetanus	16 (0.07)
		Gangrene	8 (0.04)
		Venoms	43 (0.19)
		Rabies	1640 (7.42)
**1944**	**Côte d'Ivoire**	Yellow fever	135599 (602.47)
		Tetanus	11 (0.05)
		Gangrene	8 (0.04)
		Venoms	20 (0.09)
		Rabies	580 (2.58)
**1946**	**Côte d'Ivoire**	Yellow fever	85053 (364.65)
		Meningococci	9935 (42.59)
		Tetanus	5 (0.02)
		Gangrene	6 (0.03)
		Venoms	28 (0.12)
**1947**	**Côte d'Ivoire**	Yellow fever	83397 (351.23)
**1948**	**Côte d'Ivoire**	Yellow fever	10059 (41.61)

**1949**	**Côte d'Ivoire**	Yellow fever	1808 (7.35)
**1950**	**Côte d'Ivoire**	Yellow fever	2995 (11.96)
		Tuberculosis	11048 (44.10)
**1951**	**Côte d'Ivoire**	Yellow fever	4560 (17.58)
**1952**	**Côte d'Ivoire**	Yellow fever	2033 (7.57)
	**Ghana**	Smallpox and yellow fever	Over 80,0000 (143.50)
**1953**	**Côte d'Ivoire**	Yellow fever	1761 (6.33)
		Tuberculosis	13877 (49.87)
**1954**	**Côte d'Ivoire**	Yellow fever	6775 (23.51)
		Tuberculosis	15487 (53.73)
**1956**	**Côte d'Ivoire**	Tuberculosis	22412 (72.49)
**1957**	**Côte d'Ivoire**	Tuberculosis	48603 (151.80)

Data source: [52, 56, 59].
