# Supplementary material for: The colonial response to the development of disease in Ghana and Côte d’Ivoire (ca. 1900-1955): A comparative analysis of British and French colonial health policies
Source: PLoS One. 2025 Aug 14;20(8):e0329713. doi: 10.1371/journal.pone.0329713 (PMC12352650; doi:10.1371/journal.pone.0329713)
Supplement: S20 Table — (PDF) [file pone.0329713.s020.pdf]

**S20 Table. Percentage of positive smallpox vaccinations in Côte d'Ivoire, 1925-1931.**

| Year | Number of vaccinations | Positive (%) |
|---|---|---|
| **1925** | 346,679 | 55% |
| **1926** | 408,873 | 60% |
| **1927** | 234,282 | 62% |
| **1928** | 325,418 | 75% |
| **1929** | 305,358 | 60% |
| **1930** | 320,301 | 60% |
| **1931** | 423,740 | 65% |

Data source: [57].
