# Supplementary material for: The colonial response to the development of disease in Ghana and Côte d’Ivoire (ca. 1900-1955): A comparative analysis of British and French colonial health policies
Source: PLoS One. 2025 Aug 14;20(8):e0329713. doi: 10.1371/journal.pone.0329713 (PMC12352650; doi:10.1371/journal.pone.0329713)
Supplement: S21 Table — (PDF) [file pone.0329713.s021.pdf]

S21 Table. Testing for structural breaks in smallpox vaccinations per capita.

Test	Côte d'Ivoire	Ghana
H_0: *no breaks*, H_1: $1 \leq s \leq 5$	Test statistic: UDmax(τ)= 14.38	Test statistic: UDmax(τ)= 54.39
H_0: *no breaks*, H_1: $s = 5$ HAC consistent estimator	Test statistic: supW(τ) = 2521.89, estimated break points at 1911, 1926, 1932, 1942, and 1951	Test statistic: supW(τ) = 10630.50, estimated break points at 1917, 1924, 1931, 1940, and 1947
H_0: *no breaks*, H_1: $s = 2$ HAC consistent estimator	Test statistic: supW(τ) = 1125.12, estimated break points at 1932 and 1942	Test statistic: supW(τ) = 2902.37, estimated break points at 1924 and 1947
H_0: *no breaks*, H_1: $s = 3$ HAC consistent estimator	NA	Test statistic: supW(τ) =10796.18, estimated break points at 1919, 1927 and 1947
H_0: *no breaks*, H_1: $s = 1$ HAC consistent estimator	NA	Test statistic: supW(τ) = 2380.11, estimated break point at 1924

Data source: author's own calculations.
