# Supplementary material for: The colonial response to the development of disease in Ghana and Côte d’Ivoire (ca. 1900-1955): A comparative analysis of British and French colonial health policies
Source: PLoS One. 2025 Aug 14;20(8):e0329713. doi: 10.1371/journal.pone.0329713 (PMC12352650; doi:10.1371/journal.pone.0329713)
Supplement: S22 Table — (PDF) [file pone.0329713.s022.pdf]

S22 Table. Sensitivity checks structural break tests, smallpox vaccinations per capita: substituting missing values with last available year.

**Test**	**Côte d'Ivoire**	**Ghana**
H_0: $no\ breaks$, H_1: $1 \leq s \leq 5$	Test statistic: UDmax(τ)= 17.47	Test statistic: UDmax(τ)= 60.66
H_0: $no\ breaks$, H_1: $s = 5$ HAC consistent estimator	Test statistic: supW(τ) =1429.12, estimated break points at 1912, 1926, 1933, 1942, and 1950	Test statistic: supW(τ) = 10293.96, estimated break points at 1917, 1924, 1931, 1940, and 1947
H_0: $no\ breaks$, H_1: $s = 2$ HAC consistent estimator	Test statistic: supW(τ) =1345.80, estimated break points at 1932 and 1942	Test statistic: supW(τ) = 3397.46, estimated break points at 1924 and 1947
H_0: $no\ breaks$, H_1: $s = 3$ HAC consistent estimator	NA	Test statistic: supW(τ) =10881.16, estimated break points at 1919, 1927 and 1947
H_0: $no\ breaks$, H_1: $s = 1$ HAC consistent estimator	NA	Test statistic: supW(τ) = 2614.23, estimated break point at 1924

Data source: author's own calculations.
