# Supplementary material for: The colonial response to the development of disease in Ghana and Côte d’Ivoire (ca. 1900-1955): A comparative analysis of British and French colonial health policies
Source: PLoS One. 2025 Aug 14;20(8):e0329713. doi: 10.1371/journal.pone.0329713 (PMC12352650; doi:10.1371/journal.pone.0329713)
Supplement: S23 Table — (PDF) [file pone.0329713.s023.pdf]

S23 Table. Sensitivity checks structural break tests, smallpox vaccinations per capita: using linear interpolation for missing values.

Test	Côte d'Ivoire	Ghana
$H_0: no\ breaks, H_1: 1\ \leq s\ \leq 5$	Test statistic: UDmax(τ)= 19.48	Test statistic: UDmax(τ)= 75.86
$H_0: no\ breaks, H_1: s = 5$ HAC consistent estimator	Test statistic: supW(τ) = 1593.44, estimated break points at 1912, 1924, 1932, 1942, and 1950	Test statistic: supW(τ) = 7624.59, estimated break points at 1908, 1917, 1924, 1940, and 1947
$H_0: no\ breaks, H_1: s = 2$ HAC consistent estimator	Test statistic: supW(τ) = 1693.04, estimated break points at 1932 and 1942	Test statistic: supW(τ) = 4392.25, estimated break points at 1924 and 1947
$H_0: no\ breaks, H_1: s = 3$ HAC consistent estimator	NA	Test statistic: supW(τ) =11849.44, estimated break points at 1919, 1927 and 1947
$H_0: no\ breaks, H_1: s = 1$ HAC consistent estimator	NA	Test statistic: supW(τ) = 3068.34, estimated break point at 1924

Data source: author's own calculations.
