# Supplementary material for: The colonial response to the development of disease in Ghana and Côte d’Ivoire (ca. 1900-1955): A comparative analysis of British and French colonial health policies
Source: PLoS One. 2025 Aug 14;20(8):e0329713. doi: 10.1371/journal.pone.0329713 (PMC12352650; doi:10.1371/journal.pone.0329713)
Supplement: S24 Text — (PDF) [file pone.0329713.s024.pdf]

S24 Text. Data Appendix

Disease

Data on all selected diseases for Ghana were transcribed from the Gold Coast Medical reports (1897-1955) for all available years. For 1954, no disease tables were reported. For 1926 and 1927, part of the tables were incomplete, leading to the omission of data on these years for most diseases. Disease and mortality figures for Côte d'Ivoire (1905-1940) were taken from various sources: the *Rapport Medical Annuel: Côte d'Ivoire* (1908-1912, 1914-1917, 1929-1933, 1935-1937, 1939), the *Rapport Annuel Service de Santé de la Côte d'Ivoire* (1906, 1907, 1909, 1938, 1940-1941, 1943-1944, 1946, 1948-1949) and the *Rapport Annuel d'Ensemble: Côte d'Ivoire* (1913, 1924). No (complete) information was found available for the remaining years during the selected period.

Several diseases consisted of multiple accounts in the medical reports. For instance, separate figures were reported for pulmonary tuberculosis and other types of tuberculosis in Ghana (see e.g. Gold Coast AMR, 1942, p. 7). For malaria, different types were provided according to the moment when the disease returned after an attack (Curtin, 1989, p. 133), and sometimes also included sub-types. Whenever appropriate, these subaccounts were grouped together to provide a complete figure for the disease.

Vaccination programmes

For smallpox vaccination campaigns, I transcribed figures from the Gold Coast Medical reports for Ghana for all available years (including the data used for S16 Fig). For Côte d'Ivoire, I used multiple sources. The *Rapport Medical Annuel: Côte d'Ivoire* provided information for the following years: 1907-1912, 1914-1917, 1925-1933, 1935-1937, 1939. For the remaining years, I transcribed data from the *Rapport Annuel Service de Santé de la Côte d'Ivoire* (1906,

1938, 1940-1941, 1943-1944, 1946-1952), the *Rapport Annuel d'Ensemble: Côte d'Ivoire* (1913 and 1924), and the *Rapport Annuel de la Direction de la Santé Publique* (1953-1954). I supplemented this time series with Schneider's data for the years 1942, 1945 and 1955 to 1957 [14]. To obtain per capita figures, I use population data by Frankema and Jerven [61]. For vaccinations other than smallpox (S19 Table), I used the Gold Coast Medical reports and the *Rapport Medical Annuel: Côte d'Ivoire* (1935-1937, 1939) and the *Rapport Annuel Service de Santé de la Côte d'Ivoire* (1938, 1940, 1941, 1943-1944, 1946-1954).

A note on financial years

When calculating cases per capita and vaccinations per capita, I presume that the population figures by Frankema & Jerven concern mid-year estimates, in order to obtain per capita figures for the years between 1922-1928 for Ghana. The Gold Coast Medical reports provide figures for financial years during this period, running from for example April 1, 1922 to March 31, 1923. Assuming that the population figures concern mid-year estimates allows for a calculation of per capita figures, as they concern point estimates within the reported financial years (and are not a figure concerning the full year). I use a similar reasoning for the years 1916 and 1917 for Côte d'Ivoire – when vaccination data concerns only the period until September – in order to obtain smallpox vaccinations per capita.

Mortality figures

The dataset on mortality figures, transcribed from the same sources as the data on disease, turned out more incomplete than the information on morbidity. For Côte d'Ivoire, mortality figures were only available for the years 1909 and 1931-1933 for most diseases. Information was richer for Ghana, even leading to some nearly complete timeseries (e.g. for dysentery mortality data). Given the more consistent recordkeeping, it seems plausible that (part of) the years without mortality figures actually indicate zero deaths, rather than a lack of

reporting. S25 Table to S28 Table show the ratio of deaths to cases in colonial health care facilities per disease for both countries.

Apart from the lack of data, three other observations stand out. For Ghana, (very) high death/cases ratios are found in the early years. This is mostly due to a low number of cases paired with a (nearly) equal number of deaths. For example, the report of 1902 shows three cases of lepra in colonial health care facilities, but also three deaths due to lepra, resulting in 100% percentage of deaths out of total cases. In some cases, the mortality rate even exceeds 100% of the patients, see for instance plague mortality in 1908. This is possible, because the total number of patients uses the number of incoming patients reported, to avoid double-counting patients who have remained in the hospital from previous years. However, the number of deaths reported does not allow for such a distinction, and as such, deaths include deaths from incoming and remaining patients, which allows for a death rate over 100%. These issues stop occurring for most diseases by the 1930s, when the number of (morbidity) cases start to rise as overall patient figures increased. In addition, yaws cases (Fig 2) in both countries are high, but this is not matched with high death rates (S26 Table and S28 Table). Finally, despite a high level of cases and deaths due to influenza during the 1918 influenza pandemic (S14 Fig; cases: 7756, deaths: 204) in Ghana, this does not lead to a major increase in the death/cases ratio (S27 Table).
