# Supplementary material for: The colonial response to the development of disease in Ghana and Côte d’Ivoire (ca. 1900-1955): A comparative analysis of British and French colonial health policies
Source: PLoS One. 2025 Aug 14;20(8):e0329713. doi: 10.1371/journal.pone.0329713 (PMC12352650; doi:10.1371/journal.pone.0329713)
Supplement: S25 Table — (PDF) [file pone.0329713.s025.pdf]

S25 Table. Côte d'Ivoire: total number of deaths per disease as a percentage of total cases per disease (beriberi – leprosy, rounded to two decimals).

Year	Beriberi	Chicken-pox	Dysentery	Fever	Gonorrhoea	Influenza	Jaundice	Leprosy
1909	15.00	.	47.06	.	.	.	.	.
1913	.	.	.	.	.	.	.	.
1914	.	.	.	.	.	.	.	.
1924	.	.	.	.	.	.	.	.
1928	.	.	.	.	.	.	.	.
1929	.	.	.	.	.	.	.	.
1930	.	.	.	.	.	.	.	.
1931	0.73	0.86	0.63	.	.	.	.	.
1932	.	.	0.79	.	.	.	.	.
1933	.	.	0.86	.	.	0.38	.	0.27

Data source: [57].
