# Supplementary material for: The colonial response to the development of disease in Ghana and Côte d’Ivoire (ca. 1900-1955): A comparative analysis of British and French colonial health policies
Source: PLoS One. 2025 Aug 14;20(8):e0329713. doi: 10.1371/journal.pone.0329713 (PMC12352650; doi:10.1371/journal.pone.0329713)
Supplement: S26 Table — (PDF) [file pone.0329713.s026.pdf]

S26 Table. Côte d'Ivoire: total number of deaths per disease as a percentage of total cases per disease (malaria – yellow fever, rounded to two decimals).

Year	Malaria	Measles	Plague	Sleeping sickness	Small-pox	Syphilis	Tuberculosis	Yaws	Yellow fever
1909	5.88	.	.	.	.	16.67	77.78	.	.
1913	.	.	.	.	.	.	.	.	.
1914	.	.	.	.	.	.	.	.	.
1924	.	.	.	.	.	.	.	.	.
1928	.	.	.	.	.	.	.	.	.
1929	.	.	.	.	.	.	.	.	.
1930	.	.	.	.	.	.	.	.	.
1931	.	4.76	.	.	16.67	0.18	5.26	0.01	.
1932	.	.	.	.	18.75	0.32	.	.	.
1933	.	0.29	.	0.28	.	0.32	14.29	.	.

Data source: [57].
