# Supplementary material for: The colonial response to the development of disease in Ghana and Côte d’Ivoire (ca. 1900-1955): A comparative analysis of British and French colonial health policies
Source: PLoS One. 2025 Aug 14;20(8):e0329713. doi: 10.1371/journal.pone.0329713 (PMC12352650; doi:10.1371/journal.pone.0329713)
Supplement: S27 Table — (PDF) [file pone.0329713.s027.pdf]

S27 Table. Ghana: total number of deaths per disease as a percentage of total cases per disease (beriberi – leprosy, rounded to two decimals).

Year	Beriberi	Chicken-pox	Dysentery	Fever	Gonorrhoea	Influenza	Jaundice	Leprosy
1897	.	.	4.85	.	.	.	.	.
1898	.	.	15.91	.	.	.	.	.
1899	.	.	24.14	.	.	.	.	.
1900	100.00	.	6.67	.	.	.	.	.
1901	.	.	32.56	.	.	.	.	.
1902	33.33	.	28.00	.	.	.	.	100.00
1903	12.50	.	17.86	100.00	3.70	.	.	.
1904	25.00	.	28.05	.	.	.	50.00	.
1905	26.32	.	19.82	9.09	.	.	.	.
1906	55.56	.	15.46	.	.	.	.	.
1907	9.09	.	3.46	.	0.23	.	.	0.00
1908	.	.	4.24	.	.	.	.	5.00
1909	10.53	.	4.27	.	.	.	.	.
1910	15.80	.	5.21	.	0.16	.	.	.
1911	51.61	.	5.15	.	.	.	.	.
1912	73.40	.	5.28	.	.	.	50.00	.
1913	25.42	.	3.52	.	.	.	.	1.47
1914	.	.	1.91	.	.	.	.	.
1915	70.00	.	3.38	.	.	.	.	4.08
1916	40.74	0.33	2.24	.	0.12	.	.	4.76

1917	33.33	.	1.73	0.12	0.08	.	.	.
1918	16.67	.	3.61	0.10	.	2.63	2.04	5.26
1919	37.50	.	2.62	.	.	2.96	.	.
1920	8.60	.	2.43	.	0.08	0.34	2.17	20.00
1921	23.81	.	3.66	.	.	0.54	1.45	.
01/01/1922-31/03/1922	3.13	.	1.84	.	.	.	4.76	.
01/04/1922-31/03/1923	7.27	.	2.53	.	0.08	.	.	.
01/04/1923-31/03/1924	20.00	.	3.45	.	.	.	.	.
01/04/1924-31/03/1925	14.63	.	3.17	.	0.17	0.78	1.22	1.04
01/04/1925-31/03/1926	14.49	.	2.24	.	.	0.91	4.00	0.45
01/04/1926-31/3/1927	30.30	.	.	.	0.02	.	.	.
01/04/1927-31/3/1928	.	.	.	.	.	.	.	.
01/04/1928-31/3/1929	6.67	.	2.23	2.27	0.08	0.58	1.23	0.07
1929	18.75	.	1.79	0.15	0.14	.	.	0.27
1930	9.38	.	2.73	0.40	0.07	.	2.49	0.19
1931	22.58	.	2.80	.	0.09	0.08	2.26	0.48
1932	22.22	.	3.72	1.00	0.12	0.53	1.13	0.87

1933	.	.	2.65	0.32	0.13	0.41	3.23	1.11
1934	11.11	0.17	2.68	0.80	0.08	0.61	1.58	1.01
1935	25.00	.	3.75	0.69	0.07	.	.	1.69
1936	.	0.17	3.04	0.67	0.07	.	1.31	1.40
1937	11.76	.	2.70	0.15	0.05	0.44	0.42	2.04
1938	20.00	.	2.85	0.47	0.06	.	.	1.58
1939	.	.	1.67	.	0.07	.	.	2.56
1940	1.41	.	2.77	.	0.27	0.23	.	2.19
1941	5.00	.	2.76	.	0.26	.	.	3.66
1942	10.00	.	4.96	.	0.18	.	.	3.11
1943	13.64	.	3.12	.	0.23	0.18	.	3.90
1944	15.80	.	2.43	.	0.22	0.80	.	3.42
1945	7.89	.	2.03	.	0.34	0.61	.	2.79
1946	16.67	.	2.13	.	0.27	0.60	.	2.86
1947	3.95	.	1.62	.	0.12	0.91	.	2.15
1948	2.04	.	0.99	.	0.15	0.22	.	1.78
1949	0.98	.	0.79	.	0.12	0.71	.	1.65
1950	5.00	.	1.09	.	0.06	.	.	0.18
1951	3.13	.	0.80	.	0.13	.	.	0.44
1952	0.48	.	0.20	.	0.20	0.13	.	0.47
1953	1.19	.	0.88	.	.	1.51	.	0.12
1954	.	.	.	.	.	.	.	.
1955	2.25	.	1.32	.	0.14	.	.	.

Data source: [52-56].
