# Supplementary material for: The colonial response to the development of disease in Ghana and Côte d’Ivoire (ca. 1900-1955): A comparative analysis of British and French colonial health policies
Source: PLoS One. 2025 Aug 14;20(8):e0329713. doi: 10.1371/journal.pone.0329713 (PMC12352650; doi:10.1371/journal.pone.0329713)
Supplement: S28 Table — (PDF) [file pone.0329713.s028.pdf]

S28 Table. Ghana: total number of deaths per disease as a percentage of total cases per disease (malaria – yellow fever, rounded to two decimals).

Year	Malaria	Measles	Plague	Sleeping Sickness	Smallpox	Syphilis	Tuber-culosis	Yaws	Yellow fever
1897	1.17	.	.	.	19.05	0.28	.	.	.
1898	4.24	.	.	.	10.39	.	.	.	.
1899	7.88	.	.	.	3.70	.	.	.	.
1900	5.36	.	.	.	16.57	.	33.33	.	.
1901	5.05	.	.	.	15.68	7.41	40.00	.	.
1902	5.33	.	.	.	13.52	11.54	.	.	100.00
1903	2.43	.	.	.	29.73	9.09	.	.	.
1904	1.83	.	.	83.33	1.61	.	66.67	.	.
1905	4.35	.	.	57.14	4.00	.	.	.	.
1906	1.75	.	.	100.00	5.71	18.18	66.67	.	.
1907	0.38	.	.	14.71	.	.	.	.	.
1908	0.47	.	106.19	43.75	20.22	0.29	20.00	.	.
1909	0.33	.	.	34.09	17.05	0.36	27.78	.	.
1910	0.57	2.86	.	23.08	26.06	0.26	30.00	.	90.91
1911	0.22	.	.	1.20	16.48	0.92	5.73	.	55.56
1912	0.48	.	.	17.65	14.93	0.23	9.63	.	66.67
1913	0.19	.	.	50.00	11.90	0.78	10.64	.	20.00
1914	0.37	.	.	83.33	6.25	0.89	7.72	.	50.00
1915	0.18	.	.	17.65	.	0.31	6.88	0.19	100.00
1916	0.14	.	.	37.50	.	1.03	8.83	.	83.33

1917	0.54	.	100.00	46.15	12.00	0.97	9.20	.	100.00
1918	0.41	.	.	.	.	0.95	13.81	0.23	.
1919	0.27	.	.	21.43	39.13	1.09	8.92	.	11.11
1920	0.26	.	.	11.11	24.50	0.42	10.14	0.19	33.33
1921	0.35	.	.	37.50	.	0.36	12.17	.	75.00
01/01/1922 - 31/03/1922	0.11	.	.	.	.	.	2.17	.	20.00
01/04/1922 - 31/03/1923	0.21	100.00	.	.	.	1.29	11.17	0.07	63.64
01/04/1923 - 31/03/1924	0.35	.	54.55	33.33	.	0.23	15.57	.	76.92
01/04/1924 - 31/03/1925	0.40	.	79.53	.	.	0.43	12.32	0.02	70.00
01/04/1925 - 31/03/1926	0.53	3.13	50.00	13.51	7.19	0.18	13.31	.	62.50
01/04/1926 -31/3/1927	.	.	.	.	.	0.53	13.04	.	.
01/04/1927 -31/3/1928	.	.	.	.	.	.	.	.	.

01/04/1928 -31/3/1929	1.82	.	100.00	30.95	.	0.35	8.14	.	100.00
1929	0.80	.	.	19.67	36.59	1.13	12.02	0.01	.
1930	1.12	.	.	7.00	11.43	1.18	12.33	.	50.00
1931	1.18	.	.	11.11	.	2.45	12.01	0.01	34.62
1932	1.43	.	.	6.73	.	1.49	13.28	0.01	50.00
1933	1.07	.	.	6.83	.	2.17	15.26	0.03	.
1934	0.43	.	.	5.75	.	1.70	16.23	0.02	100.00
1935	0.50	.	.	2.96	.	1.81	17.03	0.01	57.14
1936	0.50	.	.	2.78	.	3.22	15.03	0.00	100.00
1937	0.43	.	.	3.02	.	2.05	16.03	0.01	54.79
1938	0.29	1.08	.	2.97	.	1.19	17.19	0.01	60.00
1939	0.21	0.14	.	3.06	0.60	1.79	16.99	0.01	50.00
1940	0.17	.	.	2.50	.	2.15	13.02	0.00	.
1941	0.24	.	.	3.98	11.16	2.47	14.48	0.01	100.00
1942	0.26	.	.	3.22	2.48	0.79	13.75	0.01	100.00
1943	0.27	0.65	.	3.46	.	1.87	15.41	.	100.00
1944	0.21	.	.	2.74	.	2.39	13.95	0.01	.
1945	0.18	0.13	.	2.04	.	1.06	12.37	0.00	80.00
1946	0.20	0.13	.	1.71	0.27	0.54	11.35	0.00	.
1947	0.19	0.19	.	1.82	2.25	0.97	11.64	0.00	.
1948	0.19	0.19	.	1.69	4.01	0.34	8.15	0.01	.
1949	0.16	0.14	.	3.56	6.59	0.28	10.24	.	41.67
1950	0.18	0.11	.	1.53	16.46	0.55	9.57	0.00	15.38

1951	0.22	.	.	2.07	4.65	0.64	6.72	.	29.27
1952	0.13	0.03	.	1.43	3.45	0.20	7.95	0.00	100.00
1953	0.14	0.29	.	2.69	.	0.09	.	.	.
1954	.	.	.	.	.	.	.	.	.
1955	0.38	1.82	.	10.47	5.56	0.58	4.83	0.00	.

Data source: [52-57].
