# Supplementary material for: The colonial response to the development of disease in Ghana and Côte d’Ivoire (ca. 1900-1955): A comparative analysis of British and French colonial health policies
Source: PLoS One. 2025 Aug 14;20(8):e0329713. doi: 10.1371/journal.pone.0329713 (PMC12352650; doi:10.1371/journal.pone.0329713)
Supplement: S29 Text — (PDF) [file pone.0329713.s029.pdf]

S29 Text. Structural breaks testing: smallpox vaccinations per capita.

Structural break tests

To more formally assess potential trend breaks in Fig 4, I use the xtbreak package and method by Ditzen, Karavias and Westerlund to test for structural breaks [88-89]. As the number of breaks is unknown, I test $H_0: no\ breaks$ against $H_1: 1 \leq s \leq 5$, with *s* being the number of structural breaks. The findings in Table S1.4 show that H_0 can be rejected for both countries. Applying a heteroskedasticity- and autocorrelation-consistent estimator results in the same conclusion, and provides estimates for the structural breaks in both countries. These are 1911, 1926, 1932, 1942 and 1951 for Côte d'Ivoire and 1917, 1924, 1931, 1940 and 1947 for Ghana (S21 Table), when applying the maximum number of structural breaks before H_0 becomes rejected. This number equals five for both countries, using the sequential test for multiple breaks at unknown breakpoints by Ditzen, Karavias & Westerlund [89].

However, some of these estimated breaks occur around peaks in the number of smallpox vaccinations, which are not reflected in (major) changes in the trendline in Fig 4. In order to analyse this further, I limit the number of structural breaks to two for Côte d'Ivoire. This results in two estimated break points at 1932 and 1942 (S21 Table). These break points concur with changes in the trendline: around 1930 the trendline seems to break and continue at a higher level, while around 1947 the trend breaks downward (Fig 4). Based on this result, I would suggest that the estimates of 1911, 1926, and 1951 mostly pick up on a sudden peak in the number of smallpox vaccinations, while actual break points occur at the estimates of 1932 and 1947. For Ghana, applying the same method does not provide conclusive findings, as the estimated break points are not consistent when applying different criteria for the number of structural breaks (S21 Table).

As missing values are present, I opt for two sensitivity checks to see if the results remain robust. Missing values exist for Côte d'Ivoire in the years 1915, 1918-1923 and 1934. For Ghana, data is missing for 1903-1906 and 1912-1913. First, I substitute missing values with last year's value whenever possible. This does not correct for all missing values, as the value of last year was also missing in some instances. In the latter case, a missing value remains. For Côte d'Ivoire, I am able to substitute the values of the years 1915, 1918 and 1934 with last year's value. For Ghana, this transformation is used for 1903 and 1912. For Côte d'Ivoire, the results stay largely the same. Apart from a change in estimated structural break from 1911 to 1912 and from 1951 to 1950 when applying the found limit of maximum number of structural breaks (again: five), the findings remain robust when using the same tests as before (S22 Table). Based on these results, the estimated break points of 1932 and 1942 again seem to be actual structural breaks, while the others potentially pick up on smallpox vaccination spikes rather than trend breaks. For Ghana, the results also remain robust to this way of handling (part of) the missing values (S22 Table). The conclusion based on these results therefore does not deviate from the prior results.

Since the substitution method did not account for all missing values, I next opt for using linear interpolation to address the missing values. With the interpolated data included in a new time series, I again test for structural breaks using xtbreak. The first test shows that H_0 of no structural breaks can still be rejected for both countries (S23 Table), while the maximum number of structural breaks before H_0 starts to be rejected is again five according to the sequential test. For Côte d'Ivoire, the estimated structural breaks when allowing for five breaks again show two changes from 1911 to 1912 and from 1951 to 1950 (S23 Table). However, the two years that have been suggested to be the actual break points (1932 and 1942) remain consistent, and also show up when limiting the number of structural breaks to two (S23 Table). For Ghana, 1931 disappears when allowing for five structural breaks (and using a

heteroskedasticity- and autocorrelation-consistent estimator), while 1908 is estimated to be a break point (S23 Table). Limiting to fewer structural breaks leads to the same results as before (when missing values were not addressed). Therefore, it remains unclear which estimated break points for Ghana are in fact actual break points, instead of picking up sudden peaks that are not sustained trend-wise.

Interpretation

The results of the structural break tests provide evidence for the occurrence of structural breaks in both timeseries (see S31 Text). The null hypothesis of no structural breaks can be rejected, and multiple structural breaks are estimated for both countries (S21 Table). For Côte d'Ivoire, the results indicate that the estimated structural breaks at 1932 and 1942 are break points following a shift in colonial vaccination policy for Côte d'Ivoire. These concur with a rising pattern of smallpox vaccinations per capita during the 1930s, and a subsequent declining pattern in the 1940s. These findings remain robust when accounting for missing values in various manners (S22 Table and S23 Table; S31 Text).

The outbreak of more serious smallpox epidemics in Côte d'Ivoire after the initial decades of the 20th century, can (in part) explain the estimated trend break during the 1930s [14]. Descriptions in the medical reports show that vaccinations formed an important tool in trying to address smallpox epidemics (see e.g. Côte d'Ivoire RASS of 1905) [58]. As more serious smallpox epidemics started to occur (as reflected in S5 Fig), a structurally upward shift during the 1930s in the number of smallpox vaccinations administered is probable (Fig 4). Explaining the estimated structural break around 1942 is less straightforward. Potential causes include the outbreak of WWII, and a decrease in the absolute number of smallpox cases until around 1957 as shown by Schneider [14]. Moreover, S16 Fig shows that as of the 1940s, mixed smallpox and yellow fever vaccines started to become administered, which may also have contributed in the observed declining trend for this period as observed in Fig 4.

For Ghana, the results are somewhat more unclear, because the estimated structural breaks differ when applying different sensitivity checks (S21 Table - S23 Table). Despite these inconsistencies, the structural break estimated in 1924 (S21 Table) can be linked to the introduction of the Vaccination Ordinance in 1920, which can be expected to have led to an increased number of vaccinations (see also Fig 4). The medical report of 1930 also discusses that there has been an increased demand in Ashanti and the Northern Territories among chiefs and other persons in smallpox infected areas, that was met by additional vaccination efforts by the colonial administration [55]. Similarly, the estimated break point at 1947 concurs with an upward change in the trendline (Fig 4). This contrasting finding compared to Côte d'Ivoire can (in part) be attributed to colonial policy. British colonial policymakers continued to provide vaccinations against smallpox in Ghana towards the end of colonial rule, as it was deemed that 'the price of freedom from small pox is a never ending vaccination campaign' in the medical report of 1955 [56].
