# Supplementary material for: The colonial response to the development of disease in Ghana and Côte d’Ivoire (ca. 1900-1955): A comparative analysis of British and French colonial health policies
Source: PLoS One. 2025 Aug 14;20(8):e0329713. doi: 10.1371/journal.pone.0329713 (PMC12352650; doi:10.1371/journal.pone.0329713)
Supplement: S30 Text — (PDF) [file pone.0329713.s030.pdf]

# S30 Text. Glossary of diseases

This Appendix provides a short explanation of the pathology each of the diseases discussed in Sections 4.4 and 4.5.

**Beriberi.** Beriberi is a deficiency disease caused by a lack of thiamine (or vitamin B1) in daily sustenance, resulting in malnutrition [80].

**Chickenpox.** Chickenpox (or varicella) is an airborne disease caused by the varicella-zoster virus [90]. The disease is highly infectious and leads to a vesicular rash in most patients (often young children), but sometimes presents serious complications like pneumonia and even death [90].

**Dysentery.** Dysentery is an infectious intestinal disease mostly caused by either an amoeba (*E. histolytica*) or bacteria (*Shigella*), leading to bloody diarrhoea with complications such as abscesses in more serious cases [10].

**Gonorrhoea.** Gonorrhoea is a venereal disease, caused by the bacterium *Treponoma pallidum* [44]. It usually infects the throat, rectum or genitalia, and can lead to urethritis (men and women) or cervicitis in women [91].

**Influenza.** Infection by the influenza virus typically results in fever paired with muscle pain [84]. It can take on epidemic forms, spreading quickly among a population, and leading to additional deaths in elderly patients or those with existing comorbidities.

**Leprosy.** Leprosy is an infectious disease caused by the bacterium *M. Leprae* which can lead to debilitating neurological issues [92]. Historically, the disease was known for its contagiousness and the disfigurement in affected patients, and in response, patients were often isolated [93].

**Malaria.** The two main vectors for malaria are the *Anopheles gambiae* and the *Anopheles funestus* [1]. Infection by *Plasmodium* parasites (most importantly the *P. vivax* and *P. falciparum* type) carried by such vectors, leads to symptoms including fever and chills, but may also present severe complications such as anaemia and cerebral malaria [94].

**Measles.** Measles is a highly infectious disease typically occurring in childhood; symptoms include a fever, cough and a rash, but the disease sometimes poses serious complications such as blindness and pneumonia [96].

**Sleeping sickness.** Sleeping sickness, or trypanosomiasis, initially leads to fever, joint pain, headaches and swelling of the lymph nodes at the back of the neck of affected patients [6, 28]. Left untreated, the central nervous system subsequently becomes affected, resulting in lethargy or insanity, coma and eventually death. The vector is the tsetse fly, and two variants of the disease exist: a chronic version, caused by the protozoan *Trypanosoma brucei gambiense*, and an acute one caused by *T.b. rhodesiense*.

**Smallpox.** Smallpox is caused by the variola virus, resulting in symptoms such as fever and tiredness, followed by a characteristic rash in infected persons, and in some cases death (Fenner, Henderson, Arita, Ježek and Ladnyi (1988, p. 54) report infant mortality rates of over 40%) [79, 96]. The disease was declared eradicated in 1980, following a World Health Organization programme [79].

**Syphilis.** This venereal disease occurs in patients affected by the bacterium *Neisseria gonorrhoea* [44]. Left untreated, syphilis progresses along various stages from skin ulcerations (primary) to a rash (secondary) to latent syphilis (with recurring or no symptoms) or tertiary syphilis including neurological complications [97].

**Tuberculosis.** This infectious disease is caused by *M. tuberculosis*, and transmitted via the respiratory route [98]. It often affects the lungs, but can also be found in other parts of the body.

**Yaws**. Yaws is an infectious disease, caused by the bacterium *Treponema pallidum pertenue,* and leads to disfiguration, and sometimes lesions of skin and bones [99]. The disease mostly affects children, and is transmitted in warm and humid environments through direct skin contact with the infectious lesions.

**Yellow fever.** Yellow fever is transmitted by the bite of *A. aegypti* mosquitos, and causes symptoms such as fever, nausea and vomiting but also more serious complications including jaundice, haemorrhage and death [77].
