# Supplementary material for: The colonial response to the development of disease in Ghana and Côte d’Ivoire (ca. 1900-1955): A comparative analysis of British and French colonial health policies
Source: PLoS One. 2025 Aug 14;20(8):e0329713. doi: 10.1371/journal.pone.0329713 (PMC12352650; doi:10.1371/journal.pone.0329713)
Supplement: S31 Text — (PDF) [file pone.0329713.s031.pdf]

S31 Text. Extended discussion of graphs

This Appendix provides an extended explanation and interpretation of the developments shown in the graphs on official morbidity figures (per capita), for each of the selected diseases, complementing the findings in Section 4.

Yaws

Fig 2 shows that the number of yaws cases (per capita) in colonial health care facilities in Ghana is relatively low until 1925. Data on Côte d'Ivoire is only available as of 1924, but also starts out low. This is not a consequence of a small base of potential yaws patients. Forbes already deemed yaws 'an exceedingly common skin disease' in Ghana and Côte d'Ivoire in 1904 [69]. Patterson also considered the disease to be extremely common in Ghana [10]. Yaws was hyperendemic in Côte d'Ivoire during the 1910s [70]. As explained in the main text, treatment methods were limited during this early period, which explains this low and stable trend. Moreover, this trend likely also reflects the general trend of colonial health care policy focusing on European care and a limited colonial health care system during the early 20th century.

Once developments in treatment and prevention occurred in Ghana, the numbers start to change. As of the mid-1920s, the number of cases treated per capita rise in Ghana (Fig 2). This change in trend reflects medical developments explained elsewhere. The trend break also concurs with a shift in British colonial policy towards social development. Per capita figures show a relatively stable level of number of cases (per capita) treated in colonial health care facilities in Ghana from 1930 until 1940 (Fig 2). This pattern is somewhat distorted due to the use of population figures, as population grew during these years (Fig 2), but the absolute number of cases also points towards a similar plateau in the number cases treated. Section 4

discusses how issues relating to high relapse rates and reported side effects dampened progress in the 1930, which explain this stagnant trend.

During the 1940s, the trend in Ghana starts to rise, following changes in British colonial health policy, until reaching a peak at 1945 (at about 323 yaws cases in colonial health care facilities per 10,000 persons; Fig 2). Afterward, the number of cases treated (per capita) starts to decline rapidly (Fig 2), because penicillin began to be used widely by 1953. Fig 2 shows that cases almost immediately start to drop quickly following this change in treatment: from about 273 cases per 10,000 persons in 1952, to about 224 in 1953, and finally to about 42 in 1955. It should be noted that this effect is again impacted by population growth during this period, but absolute numbers of yaws cases in colonial health care facilities were also found to decrease.

For Côte d'Ivoire, Fig 2 indicates that the number of cases treated (per capita) in colonial health care facilities is lower than in Ghana for the period 1925-1935, having started from similar positions around 1925. The uptake of colonial health care services for yaws by the local population, and the organisation of treatment in Côte d'Ivoire plays a role in this difference, as explained in Section 4. After introducing a new type of treatment (see Section 4), the number of yaws cases treated (per 10,000 persons) in colonial health care facilities in Côte d'Ivoire starts to rapidly increase, and surpasses the figures for Ghana, as shown in Fig 2.

By 1940, the number of cases treated in Côte d'Ivoire reach a peak that equals over double the amount of cases per capita compared to Ghana (Fig 2, about 731 yaws cases in colonial health care facilities per 10,000 persons). The French colonial medical reports remain unclear on why the number of yaws cases rapidly start to increase between 1935 and 1940. The continued effect of a general shift in French colonial policy towards social development certainly plays a role, as a result of the expansion of the colonial health care network and increasing patient figures [66]. Moreover, another change in treatment methods occurred, as described in Section 4. Lastly, prevalence of the disease, and/or improved detection methods

may have contributed to this rapidly increasing trend, as Domergue-Cloarec describes that a considerable part of the yaws cases in French West Africa were originated from Côte d'Ivoire [42].

Fewer data is available for Côte d'Ivoire during the mid- to late 1940s, but incidence (per capita) at colonial health care facilities seems to decline rapidly in Côte d'Ivoire, and at an earlier stage than in Ghana (Fig 2). Initially, this decline is the result of a temporarily decreased capacity by the colonial health care services due to WWII [42]. Subsequently, the declining trend in Côte d'Ivoire can be explained by the widespread introduction of penicillin (similar to Ghana), especially following the start of international campaigns.

Malaria

During the initial decades of the 20th century, the official morbidity figures (per capita) for malaria in colonial health care facilities were low in Ghana and Côte d'Ivoire. As explained in Section 4, French and British colonial administrations focused on treating Europeans, which explains the low trend. This finding also fits within the general trend of early British and French colonial health care policy that considered providing health care to Europeans in the colonies its main priority, but who formed a relatively small portion of the total population, and a health system that was still being developed. Moreover, according to Patterson, hospital figures underestimated actual malaria incidence in Ghana as the local population avoided treatment for the disease from colonial health care provision [10].

Around 1930, the number of malaria cases treated in colonial health care facilities per 10,000 persons started to increase in both countries (Fig 3). Although missed at first by the colonial medical reports, the report of 1935 acknowledges the rising figures in Côte d'Ivoire. Two factors were deemed to be the cause of this changing trend: 1) increases in the incidence of malaria among Europeans and 2) a larger number of visits by malaria patients from the local

population [57]. Despite viewing malaria as ‘the greatest cause of invaliding’ and ‘one of the chief causes of sickness and ill-health’, the reports on Ghana do not acknowledge or explain why the number of patients per capita rose in Ghana around 1930 (Fig 3) [55]. However, the number of colonial health care facilities had risen substantially in both countries in the first decades of the 20th century, allowing for a greater number of patients to be treated at more locations, and colonial policy in general shifted towards social development [66].

The increasing trend continued towards the end of the colonial period, and Fig 3 shows that the number of cases treated per capita in Côte d’Ivoire is considerably higher than in Ghana. Section 4 already detailed the explanations for this observed difference elaborately, which therefore will not be repeated here.

Sleeping sickness

In the case of Ghana, low patient figures were found throughout most of the colonial period compared to Côte d’Ivoire (S2 Fig). S3 Fig separately plots the development of official morbidity figures (per capita) for sleeping sickness in Ghana, due to the large peaks in Côte d’Ivoire in the late 1930s (S2 Fig). As explained in Section 4, the low patient figures in Ghana (especially until the 1930s) were not due to a lack of prevalence of the disease, as it was widespread in the colony in endemic form. British colonial policymakers were preoccupied with the disease, and implemented several policy measures (see Section 4). By the 1930s, sleeping sickness outbreaks began to occur in Ghana, that also translated into rising figures in colonial health care facilities (S3 Fig). The implementation of several policy measures, as described in Section 4, led to a decline of official morbidity figures by the end of colonial rule, that reflected the success in containing outbreaks through British colonial health policies.

While fewer data is available for Côte d’Ivoire, S2 Fig shows an increase in sleeping sickness cases per capita that occurs nearly simultaneously to Ghana (S3 Fig), but at a much

larger scale. The inclusion of the colony of Upper Volta in 1933 inflated the number of cases treated in colonial health care facilities, and in subsequent years, the number of sleeping sickness cases treated per capita continued to rise quickly (S2 Fig). The number of cases treated (per capita) in colonial health care facilities in Côte d'Ivoire declined considerably in the 1940s, reaching levels similar to the early 1930s. Again, keeping in mind the population growth during this period, it should be noted that the decline can also be observed in absolute terms. This observed decline is (partly) due to the transfer of responsibility to another medical service (*Service Général d'Hygiene et de Prophylaxie Mobile*), which is not included in the data reported on cases treated in colonial health care facilities [59]. It also seems plausible that the difference in colonial policy between the British and French in the two colonies, as described in Section 4, also (partly) explains the observed difference in cases per capita of S2 Fig; more sleeping sickness cases were detected in Côte d'Ivoire due to the focus on treatment, while the British focus on vector control did not translate into an automatic additional detection of diseases while carrying out its preferred policy.

Yellow fever

S4 Fig shows that the overall number of cases treated for yellow fever (per capita) in colonial health care facilities was low in Ghana. For reasons other than its incidence in official morbidity records, as described in Section 4, yellow fever was nonetheless considered to be an important disease that had to be targeted by British colonial health policy.

In Côte d'Ivoire, the number of yellow fever cases treated per capita follows a similar pattern as in Ghana (S4 Fig); few cases were admitted to colonial health care facilities around the turn of the century, followed by epidemic peaks that remain comparatively low in comparison to other diseases treated in colonial health care facilities. Despite concluding that yellow fever existed throughout the full colony, the medical reports continuously report no or few cases in colonial health care facilities (see e.g. the reports of 1933 and 1938) [57]. This

pattern is likely the result of a combined effect of no available treatment, and of a quick progression of the disease in infected patients left untreated, often leading to death. Few patients would arrive on time in colonial health facilities, and even if they did, effective treatment options were not available [48].

Smallpox

The number of cases treated per capita in colonial health care facilities for smallpox are plotted in S5 Fig. The official morbidity figures (per capita) for this disease are low for Ghana and Côte d'Ivoire, as a result of a lack of an available treatment method. Preventive methods were therefore applied by British and French colonial administrators, mainly through vaccination programmes (see Section 4 for more). The peaks that can be observed in S5 Fig, reflect the epidemic nature of the disease.

Disease-specific explanations were discussed in Section 4, but several general trends contribute to an observed difference between the two colonies for five diseases (leprosy, measles, dysentery, gonorrhoea and syphilis) – with official morbidity figures in Côte d'Ivoire rising considerably as of the 1930s, while Ghana kept a lower trend. In earlier work, I show that the number of colonial health care facilities increased considerably in both countries by the 1930s, but that in per capita terms, Côte d'Ivoire had a higher number of colonial health care facilities (2.87 vs. 2.22 facilities per 100,000 persons in 1931) [66]. As a result of this expansion of the colonial health care networks, overall patient figures also increased during this period in both countries. During the 1940s, the number of health care facilities continued to rise in Côte d'Ivoire, while it declined in Ghana following the abolition of separate 'European' and 'African' facilities (Table A.1 in [66]). In previous research, I also find that the overall number of patients in Ghana continued to increase despite this change (suggesting that capacity remained equal), and perhaps more importantly, that the number of consults in colonial health care facilities in Côte d'Ivoire increased almost sixfold from 1932 to 1940 [66]. These increases

in the number of health care facilities (per capita) and the number of consults are likely driving part of the observed trend for Côte d'Ivoire for these five diseases (S6 Fig -S11 Fig.). Moreover, population grew more rapidly in Ghana compared to Côte d'Ivoire during this period (Fig 1), which increases the denominator of the morbidity per capita series. Even though the underlying data shows that the absolute number of cases was rising for dysentery, gonorrhoea, and syphilis during the 1930s and 1940s, population growth in both countries partly cancels out this change in relative terms. As explained in the main text, an alternative denominator was not available, but would have been preferable given this issue.

Leprosy

The development of official morbidity figures for leprosy is slow until the 1930s, when figures start to increase considerably following the introduction of a new French policy aimed at screening and isolation. At this time, the coastal regions were on average afflicted the most by leprosy [59]. Progress during the 1940s was hindered by a fuel shortage, nearly halting all screening practices for leprosy in Côte d'Ivoire as motorized vehicles were affected [42]. As a consequence, the number of cases treated per capita decreased considerably (S6 Fig). Moreover, the medical reports describe an unwillingness among patients to be treated on a regular basis and for longer periods of time [59].

In Ghana, the number of leprosy cases per capita treated in colonial health care facilities remain relatively stable throughout 1900-1955, apart from a sudden peak in 1950 and 1951 (S6 Fig). The medical reports do not provide an official explanation for this abrupt change, but it concurs with the mention of a new treatment method for outpatients using the drug D.A.D.P.S. in the report of 1950 [56].

Measles

Measles cases per capita (S7 Fig) also start to show a difference in trend between Ghana and Côte d'Ivoire in the 1930s; cases (per capita) in Côte d'Ivoire begin to rise, while the number in Ghana remains relatively stable. For Côte d'Ivoire, this observation is the result of epidemics, that have been discussed in more detail in Section 4. In contrast to Côte d'Ivoire, measles cases are relatively stable in Ghana throughout the period, suggesting no or few epidemics, apart from a high number of cases in 1952 (S7 Fig).

Dysentery

For Ghana, S8 Fig again shows a trend in which few patients were treated compared to Côte d'Ivoire, apart from a peak of dysentery cases (per capita) in the 1950s. Treatment against the bacillary variant became available in the 1930s with the introduction of sulpha in Ghana, in supplementation of treatment already available for the amoebic variant. However, this did not translate into rising official morbidity figures, as shown in S8 Fig. This is a contrasting finding compared to developments for e.g. yaws, for which this paper has shown a direct influence of new treatment methods on official morbidity figures. A large influx of outpatients occurred in 1952 (about 31,500 outpatients), which shows up in S8 Fig [56]. No explanation for this sudden change is provided in the medical reports, so it is unclear whether this is the result of a change in overall policy (e.g. hygiene measures) or of an outbreak of dysentery.

During the 1930s, dysentery cases per capita in colonial health care facilities in Côte d'Ivoire begin to rise (S8 Fig). As discussed in detail in Section 4, the French colonial response to epidemics of dysentery during this period plays a role, in addition to an increase following the inclusion of Haute-Volta to Côte d'Ivoire. Official morbidity figures level off after WWII (S8 Fig), which is suggested to be the result of underestimation due to a lack of observation of part of the cases [42, 59]. The report of 1949 also mentions that Ivorians tended to treat dysentery at home, and only visited colonial health care facilities in more serious cases at this time, which may have also contributed to this trend [59].

Venereal diseases

Two main venereal diseases, gonorrhoea and syphilis, are also included in the article. As discussed, for Côte d'Ivoire, the observed increase in official morbidity figures for both diseases in S9 and S10 Figs can be attributed to increased mobility [59]. After the rapid increase in the 1930s, the 1940s were characterized by a decline in cases per capita for both diseases in Côte d'Ivoire (S9 Fig and S10 Fig). The report of 1943 discusses how the reported figures do not represent the colonies' situation correctly, as syphilis was widespread but not all potential patients visited colonial health care facilities [59]. Underestimation likely also occurred for gonorrhoea. Domergue-Cloarec also points out that fluctuations in the number of cases treated in colonial health care facilities did not necessarily reflect actual changes in the prevalence of syphilis and gonorrhoea, but followed the level of attention paid to these diseases by French colonial policymakers in Côte d'Ivoire [42].

Although venereal diseases were also widespread in Ghana, this is not reflected in the number of cases per capita in colonial health care facilities (S9 Fig - S11 Fig) [55]. The diseases spread throughout the colony in the early 1900s, due to increased mobility and social change [10]. Part of the reason why this widespread prevalence of gonorrhoea and syphilis in Ghana did not translate into considerable patient figures in colonial facilities is demand-driven. The local population had been exposed to venereal diseases prior to colonial rule, and preferred to continue to rely on traditional medicine for treatment. For other British colonies, in particular Uganda (Vaughan, 1991, p. 137), the literature discusses that treatment against venereal disease through Western medicine was often provided in mission hospitals, which may have also been the case in Ghana. Vaughan also argues that British colonial administrations were reluctant to propose direct interventions in the sexual practices of the local population [13]. The availability of successful treatment in colonial health care facilities formed another contributing factor. Once antibiotics were introduced in Ghana by the British colonial administration after WWII,

they proved to be effective treatment against both gonorrhoea and syphilis in the colony, and take-up rates of colonial health care increased (also in absolute terms; S9 Fig and S11 Fig) [10].

Chicken pox

Chickenpox cases per capita are shown in S12 Fig. To some extent, they mirror the trend discussed for the previous diseases: cases (per capita) in Côte d'Ivoire are higher compared to Ghana and start to take of in the 1930s. In this respect, general development relating to the expansion of colonial health systems over time and population growth again contribute to explaining this difference. The colonial medical reports for Côte d'Ivoire do not explicitly mention this changing trend. According to the report of 1938, chickenpox cases tend to be isolated and small outbreaks occur sporadically [59]. In addition, patients are reported to often experience serious complications, and as a result nurses and auxiliary doctors sometimes confuse chickenpox and smallpox. Although not explicitly supported by the medical reports, the latter suggests a link between the development of French colonial vaccination programmes during this period (as discussed in Section 4), and the increased number of chickenpox cases treated. This also implies that it is likely that measurement error occurred in the officially recorded data for chickenpox. Moreover, Rouanet discusses how surveillance for chickenpox occurred in Côte d'Ivoire during the first three decades of the 20th century, following the imposition of French colonial health policy measures [48]. This policy may have translated into a higher official morbidity figure for the disease.

S12 Fig deviates from the group of diseases with a clear distinction based on type of colonizer, because it points towards an upward trend in chickenpox cases for Ghana in the final decades of colonial rule, rather than remaining more or less stagnant. Patterson briefly discusses chickenpox, and claims that the disease did not receive much attention from British colonial policymakers, even if the disease can be presumed to be quite common and increasingly present following increased mobility and urbanization during colonial rule [10]. Reviewing the medical

reports for the period 1930-1940 established that despite the increasing trend in S12 Fig, colonial administrators did not recognize this trend, as chickenpox was not discussed specifically. The development of smallpox vaccination programmes (Section 4.5) by British colonial administration may have contributed to the observed increasing trend: colonial attention was paid to smallpox, and in detecting and treating the disease, chickenpox cases were also found due to its similar epidemiology.

Beriberi

Beriberi cases per capita in Ghana show a relatively low and consistent number of patients per capita during the colonial period. Concurring with increased concern over the importance of nutrition for public health in Great Britain at the turn of the century, scholars showed interests in the epidemiology of beriberi and its causes in the colonies in the late 19th and early 20th century [80-82]. British colonial medical research subsequently focused their attention (in part) on analysing the distribution and determinants of beriberi as of the late 1910s. S13 Fig indicates that this research interest was not translated into increasing numbers of patients in colonial health care facilities in Ghana, likely (partly) influenced by the limited network of colonial health care facilities at the time. Despite the general widespread prevalence of the disease in Africa in the early 20th century according to Hardy, Patterson claims that beriberi was uncommon in Ghana during colonial rule [10, 80]. Based on the available data, it is impossible to verify either statement. If one is inclined to agree with Patterson, the results in S13 Fig are the result of a low prevalence, while if one agrees with Hardy, it points towards a lack of attention of British colonial policymakers in Ghana and/or lack of demand for treatment in colonial health care facilities from the local population. The upward sloping trend in the 1950s for Ghana can be linked to changes in the international environment regarding malnutrition.

S13 Fig also shows that the development of beriberi cases (per capita) in Côte d'Ivoire deviated from the trend found for part of the diseases, as it shows relatively large fluctuations and no sign of an increasing trend as of the 1930s. The medical report of 1905 considers beriberi the second most important disease (after smallpox) in Côte d'Ivoire, and links its endemic prevalence to a diet composed of mainly rice (which lacks thiamine) [58]. It was said to have taken on epidemic proportions in 1905 due to railway and port constructions in the colony. Poor quality rice rations and inadequate living conditions, combined with severe rainfalls in the wet season, led to an epidemic of beriberi among the workers [58]. Although data on Côte d'Ivoire is sparse in the remainder of the period, it seems to indicate that the number of cases were higher than in Ghana until the 1940s and experienced additional upswings (S13 Fig). As discussed previously, Slobodkin suggests that French colonial policymakers took note of the significant negative effect of malnutrition on health (particularly of labourers), but did not achieve any meaningful policy implementations following this realisation [83]. This finding may explain why the pattern of beriberi in Côte d'Ivoire developed differently than Ghana despite both displaying low figures (S13 Fig). Outbreaks occurred in Côte d'Ivoire due (in part) to changes caused by colonial rule, and despite determining the cause of beriberi in 1912, French colonial treatment methods did not become more efficient until the 1940s when vitamin B was discovered, resulting in a comparatively higher number of cases than in Ghana until the 1940s [83].

Influenza

The relevance of the inclusion of influenza among the selected diseases is motivated by the influenza pandemic that started in 1918. S14 Fig clearly shows its influence in Ghana, while unfortunately no data was available for Côte d'Ivoire. The impact of the influenza pandemic, and the British and French colonial response, has already been discussed in the main text.

Another considerable influenza epidemic occurred in 1943-1944 in Côte d'Ivoire, often paired with pulmonary complications in patients [59]. This epidemic can also be observed in S14 Fig.

Tuberculosis

Official morbidity figures for tuberculosis in Ghana show an upward trend early on. The number of cases start to really take off around 1925 (S15 Fig), which was discussed to be the result of its prevalence and of an increased take-up of treatment by the local population [54]. Mortality figures were considered to be high by British colonial administrators (see also S21 Table) compared to other diseases, as a result of local patients only seeking colonial health care in more advanced stages of the disease. Even though the numbers continued to rise (S15 Fig), it took until the mid-1930s for British colonial policymakers to recognize its threat and impose policy measures focused on prevention (see Section 4). Death rates in Ghana remained high during this period, fuelled on by low existing immunity to the disease, widespread undernourishment, and poor housing and sanitation conditions [55]. Despite the aim to prevent the disease, tuberculosis cases (per capita) in Ghana continued to remain high in the final decades of colonial rule (S15 Fig), in part due to a lack of funds and staff [56].

Data on Côte d'Ivoire is sparser, but suggests an up-and-down trend, with a generally lower number of cases treated per capita in colonial health care facilities compared to Ghana (S15 Fig). Despite showing substantial increases in official morbidity figures during the 1930s (also in absolute terms), colonial policymakers in Côte d'Ivoire failed to notice this trend at the time and only pointed out that there is little prevalence of tuberculosis in the colony (S15 Fig) [57, 59]. The number of cases treated (per capita) in the 1940s show a substantial downward trend (S15 Fig). According to Domergue-Cloarec, most cases of tuberculosis in Côte d'Ivoire followed from persons (mostly military staff) who were contaminated outside the colony, for instance in France or North-Africa (as of 1940) [42]. Since repatriations were more difficult

during WWII, the number of tuberculosis cases was lower during this time. The use of vaccinations also play a role in explaining the downward trend, as discussed in the main text.
