# Supplementary material for: The colonial response to the development of disease in Ghana and Côte d’Ivoire (ca. 1900-1955): A comparative analysis of British and French colonial health policies
Source: PLoS One. 2025 Aug 14;20(8):e0329713. doi: 10.1371/journal.pone.0329713 (PMC12352650; doi:10.1371/journal.pone.0329713)
Supplement: S32 Text — (PDF) [file pone.0329713.s032.pdf]

S32 Text. Changing determinants of disease patterns and trends, and other dilemma's

Several dilemma's pertaining to the analyses in this paper are discussed in this Appendix. Firstly, a note on determinants of the development of disease during colonial rule is warranted. It highlights several general determinants that should be kept in mind when regarding the analyses in this paper. Moreover, I motivate the choice to consider both the development of disease and the policy response by colonial administrations.

Several factors influence the general development of disease during colonial rule in African countries, and in turn the development of (potential) number of cases treated for the selected diseases in colonial health care facilities over time. Firstly, the development of colonial health care systems over time in Ghana and Côte d'Ivoire contributes to changes in disease patterns observed in colonial health care facilities, as mentioned previously. As the health care systems expanded, supply of health care widened to different regions and had the potential of treating more patients [66].

Secondly, colonial policy changed over time. Having focused on providing health care to Europeans in its colonies in the late 19th and early 20th century, British colonial policy started to widen its view towards development by the 1920s. This shift was formalized in the Colonial Development Act of 1929, which allowed for investments in areas including health care that would promote development in the colonies [67]. This programme was expanded in the subsequent decades, following the Colonial Development and Welfare Acts in 1940 and 1945. The introduction of these acts were motivated by an economic downturn in Great Britain due to World War I (WWI) and the Great Depression [65]. A similar formalized shift occurred for French colonies, signified by the introduction of the *Fond d'Investissement pour le Développement Economique et Social* (FIDES) in 1946. This fund granted subsidies to French

colonies to promote economic and social development projects [68]. Despite this comparatively late formalization of the shift towards development, French colonial policymakers in Côte d'Ivoire had already changed their stance in the 1920s, as highlighted by Lasker and Domergue-Cloarec [41-42]. France was hit economically following WWI, and Côte d'Ivoire was seen as part of the solution [41-42]. In order to profit from the economic development of the colony, investments in human capital needed to be made, causing French colonial health policy to shift from considering health care as a conquest mechanism and a necessary provision for Europeans, towards a wider introduction of policies aimed at social development. A focus on curative efforts and incidental vaccination programmes was substituted by a broadening of colonial health care policy that included the introduction of preventive and hygiene measures, and mass colonial campaigns against diseases. For Ghana and Côte d'Ivoire in particular, I show in earlier work that the network of colonial health care facilities expanded considerably over time, and that Côte d'Ivoire had a larger number of facilities with a wider geographic coverage by the end of colonial rule in comparison to Ghana [66]. Differences in British and French colonial health care policy, and their development over time, are taken into account in the interpretation of results in the remainder of this paper.

Thirdly, population grew in Ghana and Côte d'Ivoire during the first half of the 20th century (Fig 1), as discussed previously. Paired with urbanization, and developments relating to mobility and the distribution of peoples as described in the introduction, the disease environment in African countries evolved during colonial rule as diseases spread in response to these changes [2]. Lastly, as explained in Section 1, the period of colonial rule overlaps with periods of development in health care and the medical environment. The acceptance of the germ theory of disease by the start of colonial rule, advances in modern medicine, and the epidemiological transition changed the global playing field for medical care. In respect to this paper, such developments are discussed for the selected diseases if a direct link can be made.

In general, these factors should be kept in mind when regarding disease-specific developments and colonial policies over time in the remainder of this paper.

This discussion on changing determinants highlights that the development of morbidity cannot be analysed in isolation. More general dilemma's pertaining to the analyses in this research relate to the nature of health care research, and the tension between developments in disease and policy. Regarding the former, an influential issue is the lack of observing a counterfactual as illustrated by the prevention paradox. The implementation of health policy without an experimental design, especially in historical cases, automatically results in a situation in which no counterfactual can be observed. Analysing the effect of preventive measures such as vaccination is therefore not straightforward, unless a controlled experiment occurred or a natural experiment can be identified. More specific to the focus of this paper, is the tension between the development of disease and the effect of policy measures. When observing the number of cases for certain diseases in colonial health care facilities, the general disease burden in the countries is measured imperfectly, as not all cases will end up in the health care facilities (see also Section 2 on selection issues). In order to analyse and explain changes in disease patterns and trends, colonial policies also have to be considered – given their intervention in the disease environment. However, these policies were not solely implemented within colonial health care facilities, but also outside (through colonial vaccination programmes, new treatment methods etc.). This again generates noise in the analysis of the development of disease. Finally, in evaluating the effectiveness of policy measures, the question becomes whether changes in the observed number of disease cases are the effect of developments relating to population, medical advancements, colonial policy or other factors.

In the context of these dilemma's, this paper takes an approach that analyses the extent to which colonial policy measures were taken in response to the development of official

morbidity figures. It combines analyses of the number of cases in colonial health care facilities for a selected group of diseases with descriptions in primary and secondary literature. As motivated here, these two aspects form the core of the argumentation used to answer this paper's research question. Moreover, I also consider general developments whenever necessary in the interpretation of changes in morbidity in colonial health care facilities in Ghana and Côte d'Ivoire, and the way British and French colonial policymakers responded.
