# Supplementary material for: The colonial response to the development of disease in Ghana and Côte d’Ivoire (ca. 1900-1955): A comparative analysis of British and French colonial health policies
Source: PLoS One. 2025 Aug 14;20(8):e0329713. doi: 10.1371/journal.pone.0329713 (PMC12352650; doi:10.1371/journal.pone.0329713)
Supplement: S33 Text — (PDF) [file pone.0329713.s033.pdf]

S33 Text. Additional references in supporting information

87. Côte d'Ivoire. Budget du service local de la Côte d'Ivoire. 1897-1946. Located at BnF Gallica.

88. Ditzen J, Karavias Y, Westerlund J. xtbreak: Testing for structural breaks in Stata. 2020 November 19 [Cited 2024 May 2]. Available from: https://www.stata.com/meeting/switzerland20/slides/Switzerland20_Ditzen.pdf.

89. Ditzen J, Karavias Y, Westerlund J. xtbreak: Estimating and testing for structural breaks in Stata. 2021 [Cited 2024 May 2]. Github. Available from: https://github.com/JanDitzen/xtbreak/blob/main/README.md.

90. Heininger U, Seward JF. Varicella. Lancet. 2006;368(9544):1365-1376. doi: https://doi.org/10.1016/S0140-6736(06)69561-5.

91. Unemo M, Seifert HS, Hook EW, Hawkes S, Ndowa F, Dillon JR. Gonorrhoea. Nat Rev Dis Primers. 2019;5(79):1-23. doi: https://doi.org/10.1038/s41572-019-0128-6.

92. Monot M, Honoré N, Garnier T, Aroaz R, Coppée J-Y, Lacrox C, et al. On the origin of leprosy. Science. 2005;308(5724):1040-1042. doi: 10.1126/science/1109759.

93. Worboys M. The colonial world as mission and mandate: Leprosy and empire, 1900-1940. Osiris. 2000;15(1):207-218. doi: https://doi.org/10.1086/649327.

94. Miller LH, Good MF, Milon G. Malaria pathogenesis. Science. 1994;264(5167):1878-1883. Available from: https://www.jstor.org/stable/2883974.

95. Perry RT, Halsey NA. The clinical significance of measles: A review. J Infect Dis. 2004;189(Supplement_1):S4-S16. doi: https://doi.org/10.1086/377712.

96. Moore ZS, Seward JF, Lane JM. Smallpox. Lancet. 2006;367:9508:425-435. doi: https://doi.org/10.1016/S0140-6736(06)68143-9.

97. Lafond RE, Lukeheart SA. Biological basis for syphilis. Clin Microbiol Rev. 2006;19(1):29-49. doi:10.1128/CMR.19.1.29–49.2006.

98. Flynn JL, Chan J. Immunology of tuberculosis. Annu Rev Immunol. 2001;19(1):93-129.

doi: https://doi.org/10.1146/annurev.immunol.19.1.93.

99. Mitjà O, Asiedu K, Mabey D. Yaws. Lancet. 2013;381(9868):763-773. doi: https://doi.org/10.1016/S0140-6736(12)62130-8.
